# A Single‐Enzyme Activated CRISPR‐Cas12a Nano System via Subtly Balanced dsDNA for Kinetic‐Gated UDG Detection and Spatiotemporal Cellular Imaging

**DOI:** 10.1002/advs.202523400

**Published:** 2026-03-07

**Authors:** Kejun Dong, Hao Hu, Haiyun Wang, Zijia Zheng, Shuangshuang Cheng, Wan Shu, Ruisi Liu, Xiaoyan Xin, Shiyi Huang, Dingchao Qian, Xianjin Xiao, Qiang Fu, Hongbo Wang

**Affiliations:** ^1^ Department of Obstetrics and Gynecology, Union Hospital and Institute of Reproductive Health, Tongji Medical College Huazhong University of Science and Technology Wuhan China; ^2^ Department of Cardiology South China Hospital Medical School Shenzhen University Shenzhen China

**Keywords:** CRISPR‐Cas12a, DNA Nanobiotechnology, live‐cell imaging, nano‐biosensor, spatiotemporal molecular analysis, uracil‐DNA glycosylase

## Abstract

Uracil‐DNA glycosylase (UDG) is a key enzyme in base excision repair and an important biomarker for genomic stability and disease. In many reported sensing systems, uracil excision is coupled to signal generation through additional downstream BER processing steps, resulting in an indirect readout of UDG activity. Here, we report a single‐enzyme activated CRISPR‐Cas12a nanosystem driven by a subtly balanced double‐stranded DNA (dsDNA) substrate. This dsDNA serves as a kinetic gatekeeper that maintains Cas12a in an inert state until UDG‐mediated uracil excision disrupts the balance, lowering the energy barrier for crRNA invasion and initiating Cas12a trans‐cleavage. This conformationally gated mechanism directly converts a uracil excision event into an amplified CRISPR response without requiring sequential enzymatic processing. The system achieves a 1840‐fold discrimination ratio and an ultralow detection limit of 5 × 10^−7^ U/mL. Furthermore, a genetically encoded variant enables nuclear localization of Cas12a and dsDNA sensors for in situ imaging of endogenous UDG. The platform visualizes UDG dynamics across distinct cell cycle phases, realizing spatiotemporal mapping of repair activity in living cells. This work introduces a new activation paradigm for CRISPR‐Cas12a via subtly balanced dsDNA and establishes a generalizable strategy for precise molecular sensing in complex biological environments.

## Graphical abstract

Kinetic‐gated CRISPR–Cas12a activation enables single‐enzyme detection and spatiotemporal imaging of UDG

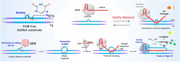

## 1 Introduction

Genomic integrity is vital for maintaining cellular homeostasis. DNA damage can lead to gene mutations, inflammation, and disruptions in cellular signaling and metabolism [1, 2, 3, 4]. Such damage is associated with the development of cancer, immune system disorders, and metabolic diseases [1, 2, 3, 4, 5]. One key mechanism for DNA repair is base excision repair (BER) [6]. Uracil‐DNA Glycosylase (UDG) is a central enzyme in this process. It recognizes and removes uracil residues from double‐stranded DNA, creating a site that allows for further repair by APE1 [6, 7]. Altered UDG expression is linked to several diseases, making it an important biomarker for conditions like cervical cancer, ovarian cancer, lymphoma, and cardiovascular injury [8, 9, 10, 11]. Therefore, there is a strong need for simple, rapid, specific, and sensitive UDG biosensors for clinical use.

Traditional UDG detection methods include mass spectrometry, radioisotope labeling, and gel electrophoresis [12, 13]. However, these techniques have significant drawbacks. They require expensive equipment, specialized personnel, and can involve radiation hazards. Additionally, they lack the sensitivity needed for clinical diagnostics. Electrochemical methods offer a more straightforward and sensitive alternative [14]. However, these methods require specialized equipment and are generally unsuitable for real‐time imaging of UDG activity in living cells. Fluorescence detection is another promising method, but it faces critical limitations. For instance, the low abundance of UDG in biological samples often requires amplification. Techniques like polymerase chain reaction (PCR), rolling circle amplification (RCA), and recombinase polymerase amplification (RPA) are commonly used [15, 16, 17], but they increase complexity, cost, and difficulty. Some enzyme‐assisted amplification methods also suffer from low sensitivity, making it challenging to detect low levels of UDG. Sun et al. developed a UDG sensor using lambda exonuclease with a detection limit of 0.0001 U/mL, but further improvement is needed to meet clinical needs [18]. A further limitation is that most UDG biosensors rely on BER‐mimetic pathways, in which uracil excision must be further processed by downstream repair enzymes before signal generation. Such multi‐step designs introduce additional reaction components, increasing operational complexity and cost. Importantly, emerging transcriptomic and clinical analyses indicate that UDG and certain downstream BER enzymes do not always exhibit parallel expression trends across different disease stages [19, 20]. In heterogeneous biological contexts, detection strategies that depend on coordinated activity of multiple repair enzymes may therefore complicate the interpretation of UDG‐associated signals. Collectively, these considerations highlight the need for a UDG biosensor that directly translates uracil excision into signal output through a mechanistically direct pathway.

In recent years, the CRISPR(Clustered Regularly Interspaced Short Palindromic Repeats)‐Cas system has revolutionized biosensing. The CRISPR‐Cas12a system, in particular, has gained attention for its strong signal amplification capability. It recognizes single‐stranded DNA (ssDNA) and generates a signal by cleaving fluorescent probes [21]. This system has been widely adopted for POCT due to its high sensitivity. Inspired by this, new tools have been developed to use CRISPR‐Cas12a for mutation detection and small molecule detection [22, 23]. However, uncontrolled trans‐cleavage activity can lead to the amplification of leakage signals, creating background noise and destabilizing the system [24]. Controlling this activity is a major challenge in developing reliable sensing platforms.

**FIGURE 1:**
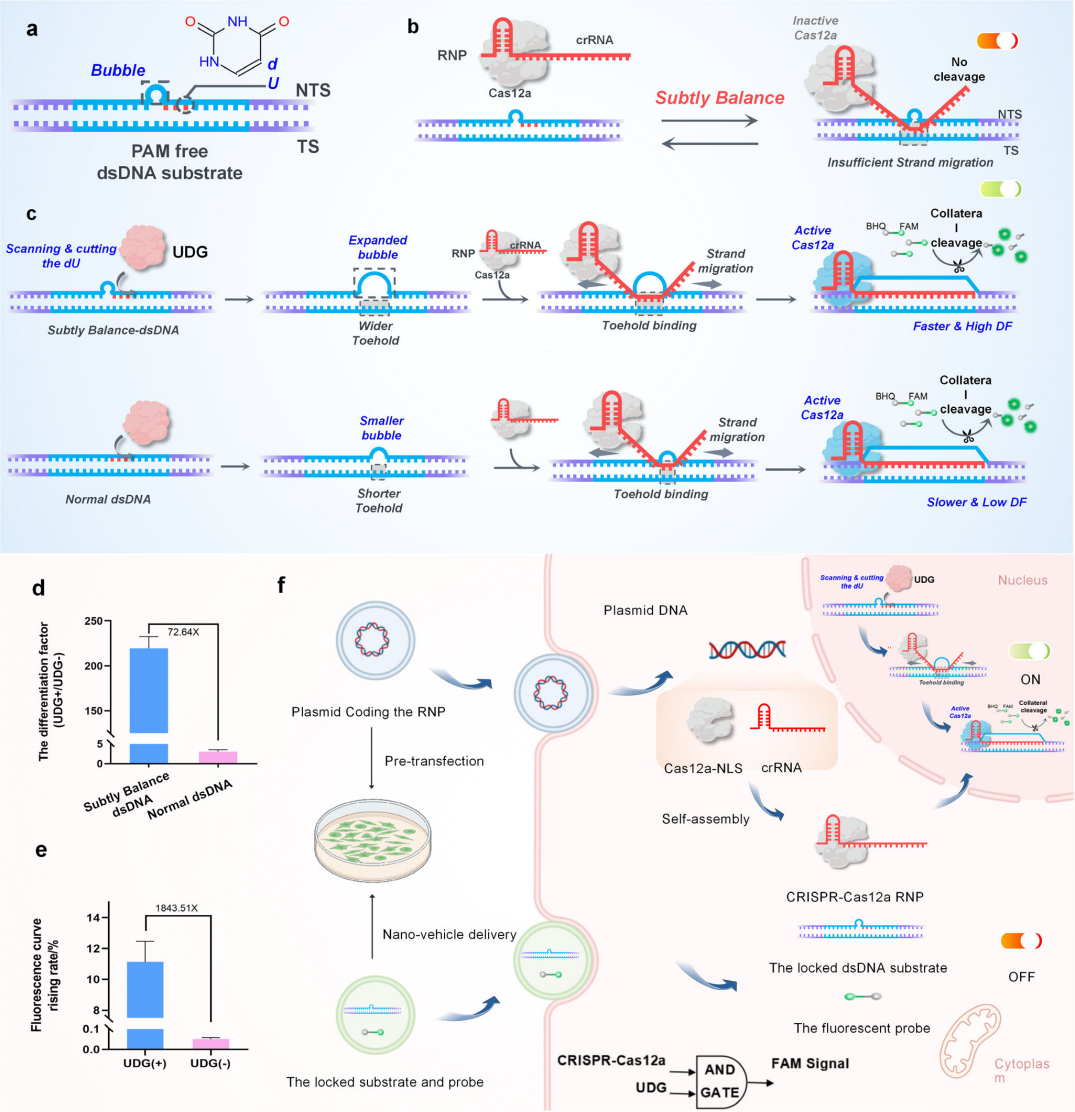
The scheme of the nano‐system. (a) The PAM‐free dsDNA substrate with the bubble and dU sites. (b) The subtly balanced substrate fails to trigger Cas12a activation without UDG. (c,d) The contrast of the subtly balanced dsDNA substrate and the normal one. After UDG treats a subtly balanced substrate, the wider toehold provides to crRNA‐DNA hybridization reaction with faster reaction and high DF by active Cas12a. (e) The contrast between UDG(+)/UDG(‐) of a subtly balanced substrate. (f) The scheme of the intracellular imaging. After the nano‐system delivers to the cell, UDG and Cas12a will construct a system for spatiotemporal cellular imaging.

In this study, we engineered a PAM‐free, subtly balanced dsDNA substrate to regulate CRISPR–Cas12a activation through kinetic gating. The designed duplex incorporates a uracil‐containing site adjacent to a defined bubble structure (Figure 1a,b), serving as a structural switch for UDG recognition. In the absence of UDG, the CRISPR‐Cas12a system remains inactive (Figure 1b). Upon UDG‐mediated uracil excision, local destabilization of the duplex lowers the kinetic barrier for strand invasion, enabling productive crRNA propagation and triggering Cas12a trans‐cleavage (Figure 1c). This conformationally gated mechanism directly converts a uracil excision event into catalytic signal amplification without requiring sequential enzymatic processing. Through systematic optimization of uracil positioning, substrate architecture, and ribonucleoprotein (RNP) concentration, the system achieves a detection limit of 5 × 10^−7^ U/mL and a 1840‐fold difference (Figure 1e) in reaction rates between the absence and presence of UDG. For intracellular application, we combined a genetically encoded Cas12a–crRNA module with a nano‐delivered dsDNA recognition substrate and fluorescent reporter (Figure 1f). Following nuclear localization of Cas12a, the system responds to endogenous UDG activity and generates a spatially confined fluorescence signal. This configuration enables visualization of UDG dynamics across distinct cell‐cycle phases, demonstrating spatiotemporal monitoring of DNA repair activity in living cells. In summary, this work establishes a kinetically gated, single‐enzyme–triggered strategy for UDG sensing and expands the mechanistic framework for CRISPR‐based molecular diagnostics.

## 2 Results and Discussion

### 2.1 Construction of a CRISPR‐Cas12a‐Mediated Subtly‐Balanced dsDNA‐Based UDG Biosensor

We developed a novel UDG biosensor that operates without the need for BER enzyme assistance. The system is based on a simple structure consisting of a subtly balanced double‐stranded DNA (dsDNA) substrate, which lacks PAM sequences, CRISPR‐Cas12a ribonucleoprotein (RNP), and a FAM/BHQ‐labeled single‐stranded DNA (ssDNA) fluorescent probe. The dsDNA substrate is pre‐configured with a small mismatch bubble, which we define as the “subtly balanced” state. Adjacent to this bubble, we incorporated a deoxyuridine (dU) site (Figure 1a; Table S1). In the absence of UDG, the subtly balanced dsDNA substrate prevents crRNA hybridization due to the lack of an invasion site, and the CRISPR‐Cas12a system remains inactive (Figure 1b). However, in the presence of UDG, the enzyme scans the substrate and cleaves the dU site to generate an abasic (AP) site. This action enlarges the mismatch bubble (Figure 1c), exposing the crRNA invasion toehold, reducing the kinetic barrier for strand invasion, and disrupting the balance. The crRNA then undergoes strand transfer with the dsDNA substrate, activating CRISPR‐Cas12a without requiring additional enzymes. This results in cleavage of the fluorescent probe and the generation of a reporter signal. By exploiting the strong trans‐cleavage activity of CRISPR‐Cas12a, the system achieves highly sensitive and specific detection of UDG with minimal background interference, eliminating the need for external enzyme assistance.

### 2.2 Validation of System Feasibility and Preliminary Optimization

We first evaluated the low‐background characteristics of the subtly‐balanced dsDNA substrate. Initially, we prepared the dsDNA using r2.1 Buffer, a commonly used buffer for constructing CRISPR‐Cas12a‐based POCT platforms. As shown in Figure S1a, compared to the positive signal from single‐stranded DNA (ssDNA), the suppression of leakage in the dsDNA substrate was inadequate. Even when the non‐template strand (NTS) ratio was increased to 4×, the discrimination from the positive control reached only 15.4‐fold (Figure S1b), which was insufficient to meet the low‐background requirements for clinical applications.

**FIGURE 2:**
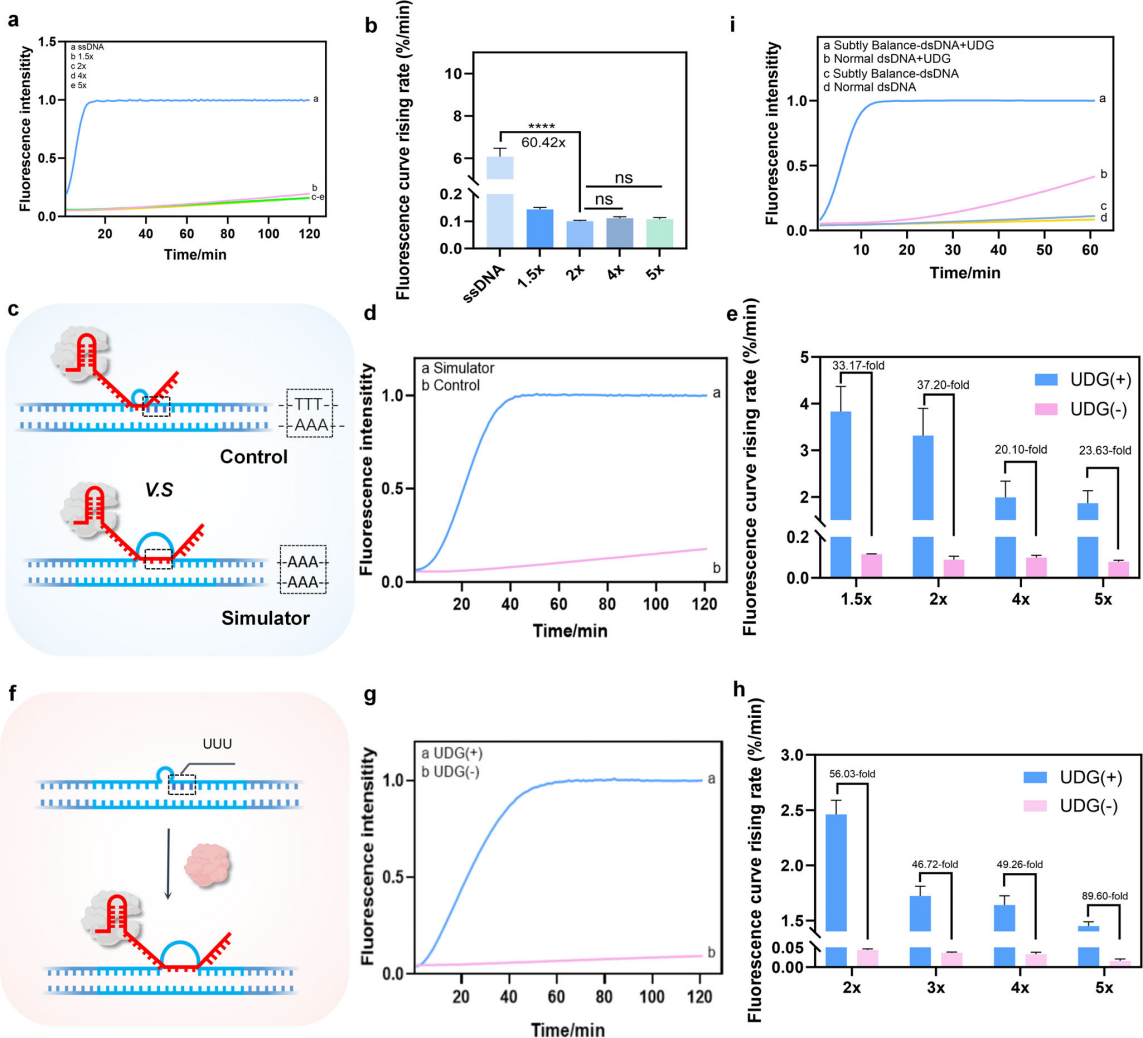
Validation of System Feasibility. (a,b) The leakage evaluation under different concentrations of the NTS strand. ^****^, *p* < 0.0001; ns, *p* > 0.05. (c–e) Mismatch strand to simulate UDG treatment. The fluorescence curve and rising rate for different concentrations of the NTS strand (2×, d; 1×, 100 nm). (f–h) The fluorescence curve and rising rate for different concentrations of the NTS strand(2×, g) under UDG treatment (5 × 10^−3^ U/mL). (i) The comparison of a subtly balanced dsDNA substrate and the normal substrate for UDG detection. *n* = 3.

To improve the suppression of leakage, we referenced the approach by Xu et al. and replaced r2.1 Buffer with Thermopol Buffer during the preparation of the dsDNA substrates [29]. As shown in Figure 2a, using Thermopol Buffer significantly enhanced the performance of the subtly‐balanced dsDNA substrates. At an NTS: TS ratio of 4:1, the discrimination improved to 54.6‐fold. Next, we optimized the NTS concentration. We tested various NTS concentrations (1.5×, 2×, 4×, and 5×; 1×, 100 nm) and analyzed the resulting system discrimination. As shown in Figure 2b, when the NTS strand was set to 2×, the relative reaction rate compared to ssDNA increased to 60.4‐fold. Notably, further increasing the NTS concentration beyond 2× did not result in a significant improvement in discrimination. These results indicate that the TB‐dsDNA substrate demonstrates low‐background characteristics, with optimized conditions for system performance, paving the way for further refinement and clinical applicability.

Furthermore, we used mismatched dsDNA, as shown in Figure 2c, to simulate the disruption of the subtle equilibrium following UDG treatment. We also evaluated the impact of NTS concentration on detection performance under these conditions. As shown in Figure 2d,e, and Figure S2, when the NTS was set to 1.5×, 2×, 4×, and 5×, the system's discrimination between the two substrates was 33.17, 37.20, 20.10, and 23.63, respectively. Next, we substituted the mismatched thymine (T) in the dsDNA with uracil (U) and assessed the system's ability to recognize UDG at a reaction concentration of 5 × 10^−3^U/mL (Figure 2f). In the absence of APE1/Endo IV assistance, the system responded efficiently to UDG. We then adjusted the proportion of NTS labeled with U. At an NTS ratio of 2×, the system achieved optimal discrimination, with a 56.03‐fold difference (Figure 2g; Figure S3). Notably, the UDG concentration used in this experiment was 5 × 10^−3^ U/mL, which is near the detection limits of most conventional methods. Under these conditions, the system reached a plateau approximately 40 min after reaction initiation, indicating its high efficiency and stability.

Next, we compared the UDG sensing capabilities of conventional dsDNA substrates with those of subtly‐balanced dsDNA substrates to highlight the necessity of using the latter. Although conventional dsDNA substrates without mismatches showed lower leakage levels, they exhibited slower reaction rates and reduced discrimination during UDG sensing (Figures 2i and 1d). We hypothesize that the conventional substrates are more structurally stable due to the absence of mismatched regions. As a result, the toehold formed after UDG treatment for crRNA chain migration is shorter, making the reaction more difficult compared to the subtly‐balance dsDNA substrates. To further investigate this, we simulated the reaction using DNA chains and performed computational modeling with NUPACK [25, 26]. The results showed that the ΔG (free energy change) for the reaction in the subtly‐balance dsDNA group is negative, indicating that the reaction is thermodynamically favorable. In contrast, the reaction in the conventional dsDNA group could not proceed spontaneously (Figure S4), providing additional support for our hypothesis.

**FIGURE 3:**
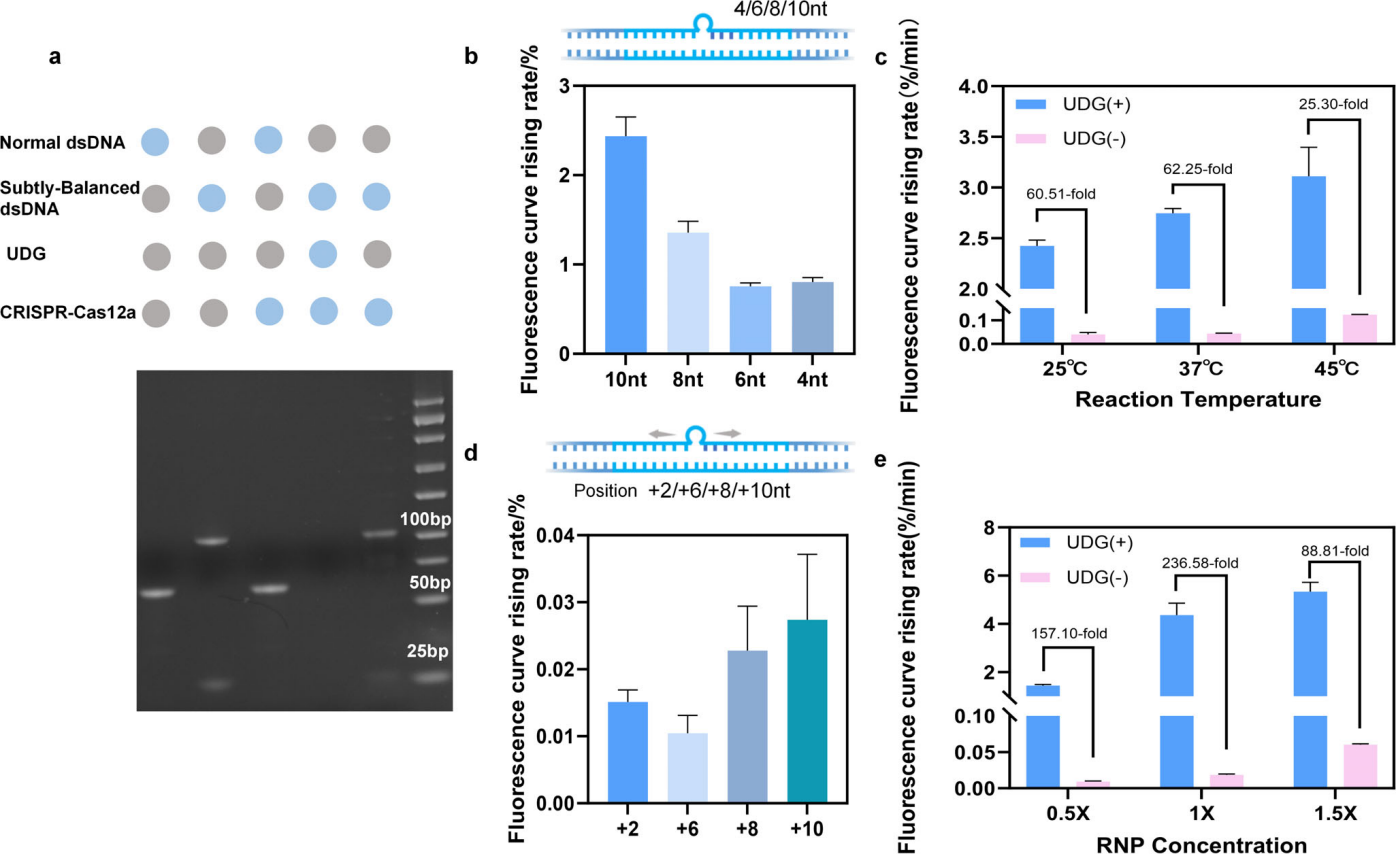
Preliminary Optimization of the system. (a) Native PAGE analysis of Cas12a reacting with subtly balanced double‐stranded DNA substrate. (b) The optimization of the bubble size. (c) The DF for the reaction temperature. (d) The optimization of the bubble location. (e) The DF for the RNP concentration. 1×, 17.2 nm. *n* = 3.

Subsequently, we validated the system using gel electrophoresis. We first examined the reaction of crRNA with subtly‐balanced dsDNA. As shown in Figure S5, the PAGE results indicate that crRNA can hybridize with the substrates, resulting in the development of larger molecular bands. It should be noted that the migration position of the subtly‐balanced dsDNA substrate was higher than the theoretical double‐stranded position. This may be related to the pre‐designed bubble structure and single‐stranded tail structure, which alter the friction and charge/friction ratio of the molecule within the gel pores, slowing its migration rate [30, 31]. Furthermore, we observed the reaction of the dsDNA substrates with CRISPR‐Cas12a. As shown in Figure 3a, three reaction groups were prepared: (1) subtly‐balanced dsDNA substrate; (2) subtly‐balanced dsDNA substrate with Cas12a; (3) subtly‐balanced dsDNA substrate with UDG and Cas12a; and (4) the control groups. The results showed that the subtly‐balanced dsDNA substrate alone could not activate the trans‐cleavage activity of Cas12a. Distinct ssDNA bands remained after the reaction, confirming the system's low background as previously described. In contrast, upon addition of UDG, the balance of the dsDNA substrate was disrupted, leading to activation of the CRISPR‐Cas12a system.

Next, we investigated the effects of bubble position and base count on system performance. As shown in Figure 3b and Figure S6, the bubble size was sequentially set to 4, 6, 8, and 10 nucleotides to evaluate changes in reaction rate. Within this range, variations in bubble size had minimal influence on background leakage, whereas larger bubbles enhanced signal discrimination. Therefore, a 10‐nucleotide bubble was selected for subsequent experiments. We also varied the bubble position (Figure 3d) to assess its effect on the background signal. As shown in Figure 3d and Figure S7, the strand positioned at +6 (Table S2) exhibited the most effective leakage suppression. Optimization of the Cas12a RNP loading amount and reaction temperature was then performed. Reaction temperatures of 45°C, 37°C, and 25°C were tested to evaluate thermal effects on the UDG sensing system. As shown in Figure 3c and Figure S8, discrimination values at these temperatures were 60.51, 62.25, and 25.30, respectively, indicating that 37°C was optimal. Preliminary optimization of RNP concentration was further conducted at 0.5×, 1×, and 1.5×. The corresponding discrimination values were 157.10, 236.58, and 88.81 (Figure 3e). Although increasing the RNP concentration to 1.5× raised material costs, the system reached a plateau within approximately 20 min, significantly reducing reaction time (Figure S9). These results indicate that multidimensional optimization is essential for achieving a balance between specificity and sensitivity in biosensor construction. Accordingly, an orthogonal optimization strategy was employed in subsequent experiments to further explore its impact on detection limits.

### 2.3 Orthogonal Matrix Optimization and Evaluation of System Specificity and Sensitivity

**FIGURE 4:**
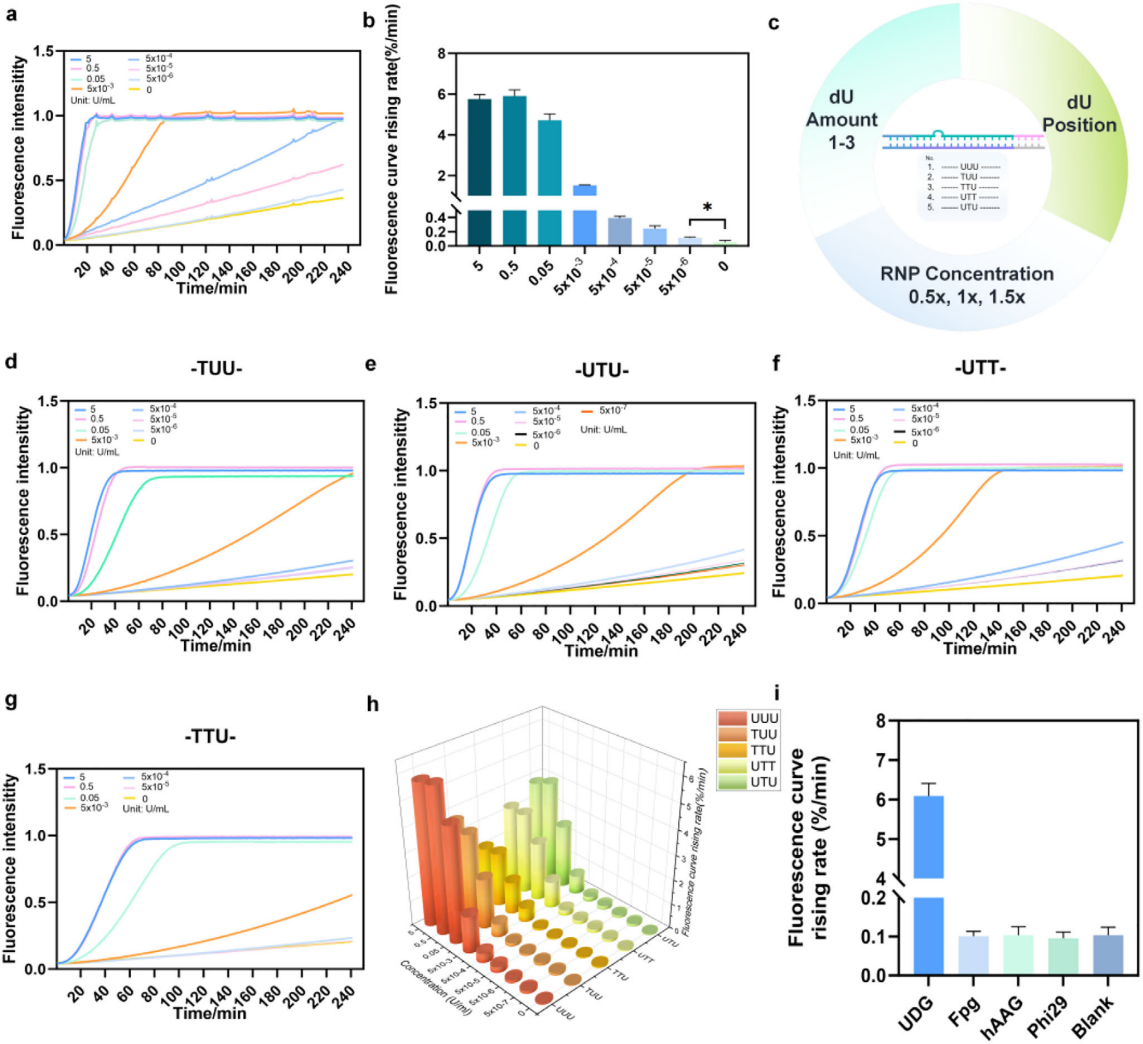
Orthogonal Matrix Optimization and Evaluation of System Specificity and Sensitivity. (a,b) The limit of detection analysis for strand TS‐1(UUU). ^*^, *p* = 0.0253. (c) The scheme of Orthogonal Matrix Optimization for dU amount, position, and RNP concentration. (d–g) The LOD analysis for different NTS strand groups. (h) The analysis for RNP concentration of 1× (17.2 nm). (i) The specificity analysis of the system. *n* = 3.

We first evaluated the sensitivity of UDG detection using dsDNA substrates (NTS‐UUU/TS‐1, Table S1 and S3). As shown in Figure 4a,b, the system effectively distinguished 5 × 10^−6^ U/mL from the blank within 160 min. However, as previously discussed, factors such as the number and position of uracil sites, as well as RNP concentration, can affect detection performance and construction cost. Adjusting each factor individually would greatly increase optimization time and make it difficult to determine the optimal configuration. To address this, we performed orthogonal matrix optimization involving these three parameters to identify a balanced design that optimized cost, specificity, and sensitivity.

As shown in Figure 4c, five NTS strands were designed: UUU, TUU, UTU, TTU, and UTT (Table S3). Orthogonal experiments were then conducted using RNP concentrations of 0.5×, 1×, and 1.5×. At an RNP concentration of 1×, the system containing a single uracil site could distinguish the blank from 5 × 10^−5^ U/mL within 100 min (Figure 4f). Incorporating two uracil sites (Figure 4d,e) also enabled accurate detection of low UDG concentrations. Notably, the UTU configuration achieved clear discrimination at 5 × 10^−7^ U/mL within 160 min (Figure 4e; Figure S10a). Compared with existing fluorescence‐based assays (Table S6), this system achieved UDG sensing at the 10^−7^ U/mL level without requiring APE1 or Endo IV, representing a 1–3 order‐of‐magnitude improvement in sensitivity [15, 16, 17, 18]. We further examined the detection performance of all five TS strands under different conditions. As shown in Figure 4h and Figure S10, the UUU strand consistently displayed the highest reaction rate. When the number of uracil bases was reduced to two, a slight decrease in reaction rate was observed, but the residual instability after UDG treatment remained sufficient to activate Cas12a. Increasing RNP concentration to 1.5× markedly shortened the reaction time. As shown in Figure S10c and S10d, the optimized system achieved a detection limit of 5 × 10^−6^ U/mL within 60 min, demonstrating excellent efficiency. Collectively, the orthogonal optimization strategy enabled comprehensive evaluation of multiple parameters, maximizing the system's performance and identifying its optimal configuration. These findings highlight the strong adaptability and tunability of the platform, allowing a flexible balance between construction cost and analytical sensitivity. This approach provides new insights and strategies for the rational design of next‐generation UDG biosensing tools.

Furthermore, we analyzed the specificity of the system response. We compared the system's responses to 8‐oxo‐guanine DNA glycosylase (Fpg), bovine serum albumin (BSA), Phi29 polymerase, blank controls, and UDG. As shown in Figure 4i, the system exhibited a specific response only to UDG, with no detectable signals for other glycosylases or interfering proteins. These results confirm the system's excellent response specificity.

### 2.4 Analysis of UDG Activity in Cell Lysates Amidst APE1 Knockdown

UDG activity is closely associated with the prognosis of multiple malignant tumors (Figure S11). Therefore, monitoring UDG activity in complex biological environments using APE1‐independent systems is essential for accurate disease evaluation. As shown in Figure S12, analysis of gastric adenocarcinoma data from the TCGA database reveals significant differences in APE1 expression across disease stages, with markedly lower APE1 levels in stage II, III, and IV patients compared to stage I patients. In contrast, UDG expression remains relatively stable. These findings suggest that UDG and certain downstream BER enzymes may not exhibit parallel expression patterns during tumor progression. In such heterogeneous biological contexts, detection strategies that depend on coordinated activity of multiple repair enzymes may introduce variability into signal interpretation. Therefore, a sensing strategy that directly translates uracil excision into signal output may offer a more reliable means of assessing UDG activity in heterogeneous disease contexts.

**FIGURE 5:**
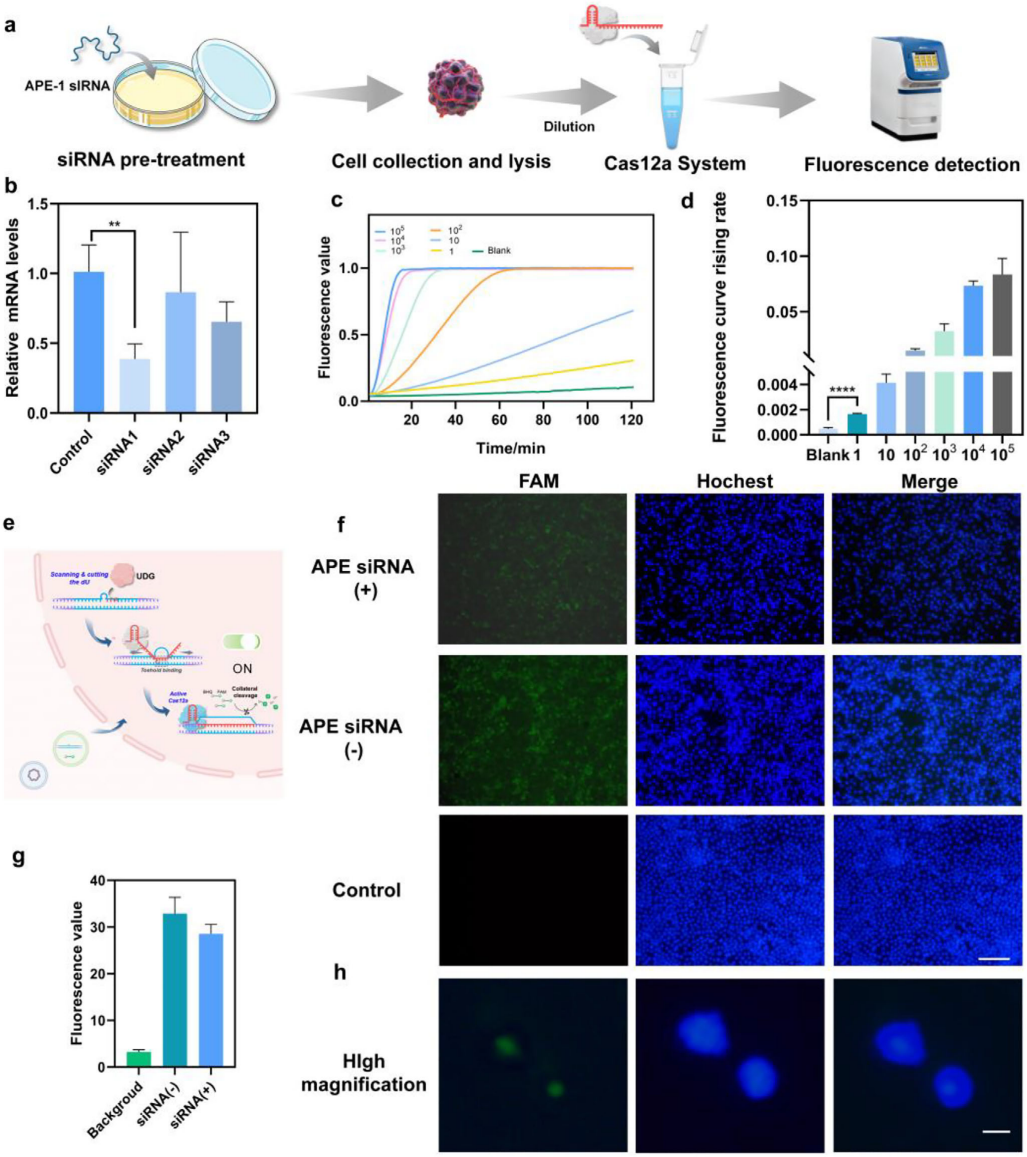
Sensing of UDG Activity in Cell Lysates and Intracellular Imaging Mediated by Nanoparticle Delivery. (a) The scheme for lysate analysis. (b) Evaluation of the effect of the siRNA. ^**^, *p* = 0.0081. (c,d) The LOD analysis of the lysates. ^****^, *p*<0.0001. (e) The scheme of intracellular imaging of the nano system. (f) Fluorescence photos of the nano system under the condition of APE siRNA(+)/APE siRNA(−), scale bar, 50um. (g) Relative fluorescence value of the photos. (h) High magnification fluorescence photos for the system, scale bar, 10 um. *n* = 3.

We first evaluated UDG activity in HeLa cell lysates (Figure 5a). To eliminate potential interference from endogenous APE1, HeLa cells were pretreated with APE1‐specific siRNA (Figure 5a). Three siRNA sequences targeting APE1 were designed and assessed for knockdown efficiency. As shown in Figure 5b, siRNA‐1 exhibited the strongest suppression of APE1 mRNA expression and was therefore selected for subsequent experiments. After confirming successful APE1 knockdown, the pretreated cell lysates were analyzed. Even in the absence of APE1 interference, the system rapidly and specifically detected UDG activity within 10 min (Figure 5c,d). These findings confirm that the biosensor retains high sensitivity and stability in complex biological environments, demonstrating strong robustness and establishing a solid foundation for subsequent intracellular UDG imaging studies.

### 2.5 Spatiotemporal Imaging of Intracellular UDG Activity with an APE1‐Independent CRISPR‐Cas12a System

Intracellular UDG plays a critical role in DNA damage repair and mismatch correction, making its in situ imaging essential for evaluating DNA repair dynamics. To achieve this, plasmids encoding Cas12a fused with a nuclear localization sequence (NLS) and the corresponding sgRNA were constructed in advance. Following transfection, the expressed Cas12a protein and sgRNA pre‐assembled into active complexes and entered the nucleus under NLS guidance (Figure 5e). To deliver the sensing components, Lipo8000 was used to encapsulate the subtly‐balanced dsDNA substrates and fluorescent probes, forming a nano‐delivery vehicle. Once the Cas12a–sgRNA complex accumulated in the nucleus, the arrival of the substrate allowed the system to respond to endogenous UDG activity. UDG‐mediated cleavage disrupted the subtly equilibrium of the dsDNA substrate, thereby activating the CRISPR‐Cas12a trans‐cleavage reaction and generating a detectable signal.

In complex biological environments, factors such as non‐specific nucleases and intracellular transport accessibility all impact the functionality of imaging systems [32, 33]. Based on the established design principle, we first assessed the delivery efficiency of the nano‐vehicle. Fluorescent DNA strands labeled with FAM were used as markers. As shown in Figure S13, fluorescence imaging confirmed that the nano‐vehicle achieved efficient intracellular delivery. Meanwhile, we also evaluated the potential for dsDNA to enter the cell nucleus. We performed additional experiments using a FAM‐labeled subtly balanced dsDNA substrate under the same transfection conditions as those used for cellular imaging. Cells were counterstained with Hoechst to visualize nuclei. The microscopy revealed clear colocalization of the green FAM signal with the Hoechst‐stained nuclear regions (Figure S14). It should be noted that, unlike NLS‐Cas, we recognize that dsDNA substrates are unlikely to actively enter the nucleus. Instead, they may pass through the nuclear pore complex via size‐dependent diffusion and enter the nucleus after escaping from endosomes [34, 35]. Once inside the nucleus, substrates undergo UDG processing to reach the threshold for triggering Cas12a, thereby generating a signal. Previous reports on CRISPR‐mediated activation of intranuclear nucleic acid probes further validate the feasibility of this mechanism in living cells [36].

Furthermore, to exclude potential interference from endogenous APE1, we applied siRNA‐mediated knockdown as described in Section 2.4. Even after APE1 suppression, the sensor maintained its ability to produce strong FAM fluorescence signals within cells, which colocalized with the Hoechst‐stained nuclei (Figure 5f). It is particularly noteworthy that certain nuclear subtypes of UDG, such as SMUG1, are closely associated with DNA damage repair in the cell nucleus. Recent studies suggest that these subtypes may participate in DNA damage repair alongside PARP [7], potentially serving as effective biomarkers for PARP inhibitor‐related targeted therapy monitoring. Besides, following siRNA treatment, the overall fluorescence intensity of the cells decreased slightly (Figure 5g). Intracellular APE1 knockdown partially slowed AP site processing, leading to a discernible intensity reduction when directly compared. Notably, under conditions where UDG and APE1 levels vary across disease progression stages, conventional UDG assays may yield false negatives when APE1 levels are low. The system eliminates this bias by isolating APE1 effects, thereby reducing false‐negative results.

**FIGURE 6:**
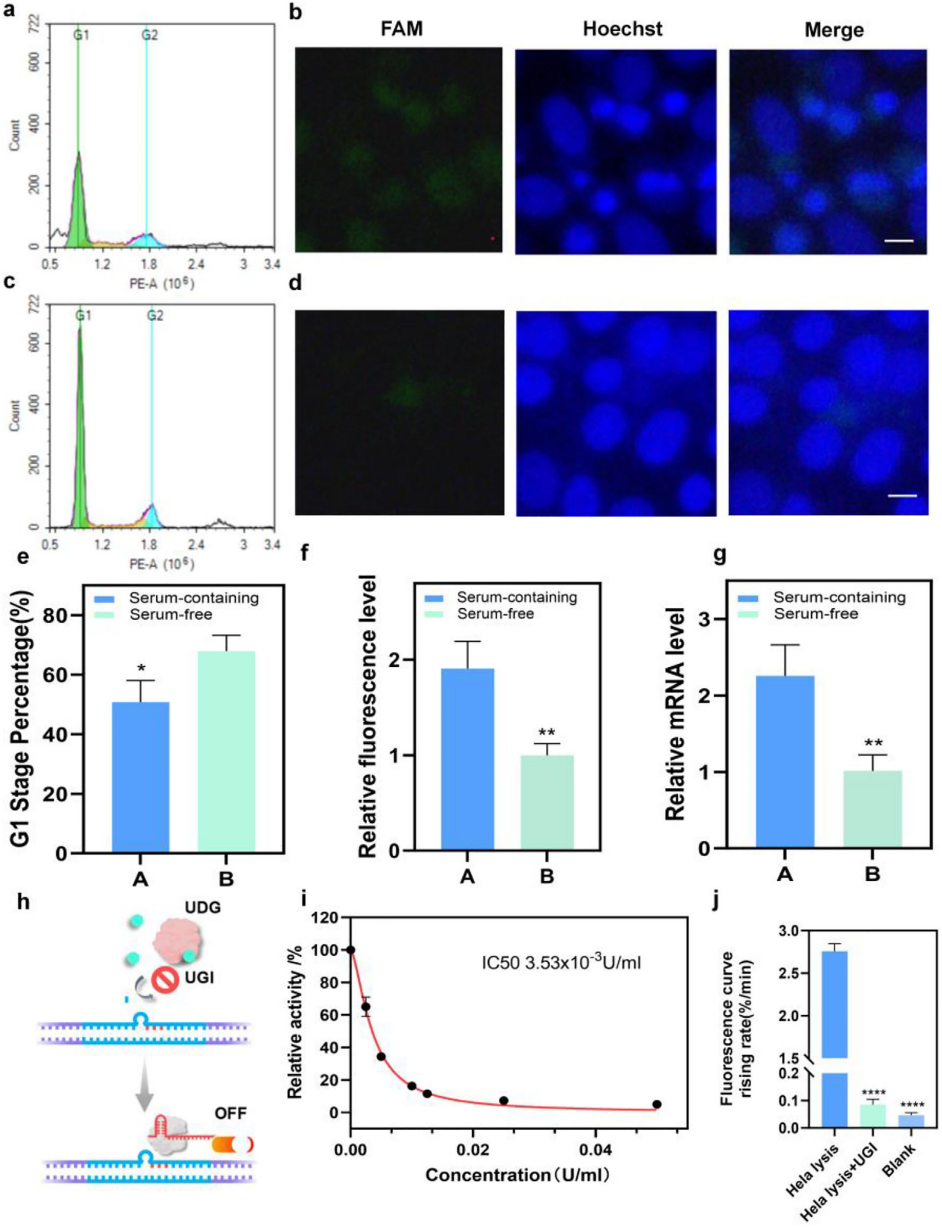
Intracellular UDG Activity Imaging Across Different Cell Cycles and the inhibitor scanning. (a,b) Cell cycle analysis of the serum‐containing medium group and the fluorescence photos of UDG activity. Scale bar, 10 um. (c,d) Cell cycle analysis of the serum‐free medium group and the fluorescence photos of UDG activity. (e) The G1 stage percentage after the treatment. ^*^, *p* = 0.0310. (f) The relative fluorescence level of the photos. ^**^, *p* = 0.0071. (g) The relative UDG mRNA level by qRT‐PCR of the treatment. ^**^, *p* = 0.0094. (h) The scheme of UGI. (i) The relative activity of UDG under the treatment of different UGI concentrations. (j) The fluorescence curve rising rate for the cell lysis and the lysis with UGI. ^****^, *p* < 0.0001. *n* = 3.

We further examined the system's imaging capability across different cell cycle stages. Cells were synchronized by serum starvation [27], and cell cycle distribution was analyzed. Starved cells displayed a dominant G1‐phase peak (Figure 6c,e), consistent with a quiescent state, whereas the control group showed a broader distribution (Figure 6a). Correspondingly, fluorescence imaging revealed markedly reduced signals in the starved group compared with the control (Figure 6b,d,f). At the same time, we analyzed the mRNA levels of UDG in cells from different treatment groups to provide corroborating evidence. As shown in Figure 6g, UDG levels in cells from the starvation‐treated group decreased compared to the control group. These findings demonstrate that the developed sensing system can effectively monitor UDG activity variations across cell cycle phases, providing a new approach for CRISPR‐based intracellular enzyme activity analysis.

### 2.6 Scanning of UDG Inhibitor

Targeting DNA damage repair pathways has become a major focus in tumor‐targeted therapy. As a key enzyme in base excision repair, UDG represents an important therapeutic target. Accordingly, sensitive monitoring of UDG inhibitor efficacy is critical for developing diagnostic and drug‐screening tools. We selected uracil‐DNA glycosylase inhibitor (UGI) as a model compound [28], which suppresses UDG activity through 1:1 binding (Figure 6h). UDG was maintained at a concentration of 5 × 10^−^^4^ U/mL, and a gradient of UGI concentrations was used to assess inhibition efficiency. As shown in Figure 6i, the fluorescence response rate decreased progressively with increasing UGI concentration, indicating dose‐dependent inhibition. Data fitting yielded an IC50 value of 3.53 × 10^−3^ U/mL (Figure 6i). To further verify system performance in complex biological environments, we tested UGI inhibition in cell lysates. As shown in Figure 6j, the addition of UGI markedly decreased the fluorescence intensity relative to the untreated lysate, confirming that the system can reliably detect UGI activity under biologically relevant conditions.

## 3 Conclusion

In this study, we established a single‐enzyme activated UDG sensing system based on a subtly balanced dsDNA substrate that regulates CRISPR‐Cas12a activation through kinetic gating. The engineered duplex maintains Cas12a in an inactive state under basal conditions, while UDG‐mediated uracil excision induces local structural destabilization that lowers the kinetic barrier for productive strand invasion, thereby initiating Cas12a trans‐cleavage and catalytic signal amplification. Through orthogonal optimization, the biosensor achieved high sensitivity, distinguishing 5 × 10^−6^ U/mL UDG within 60 min and 5 × 10^−7^ U/mL within 160 min‐detection limits 1–3 orders of magnitude lower than existing systems. The platform also demonstrated strong performance in single‐cell UDG sensing and intracellular spatiotemporal imaging, confirming its robustness and reliability. This enzyme‐independent tool reduces reliance on the BER pathway, lowers fluorescence detection costs, and offers a new approach for visualizing true intracellular UDG activity. The biosensor design effectively balances recognition specificity with response sensitivity, providing a promising foundation for clinical diagnostics and expanding the potential of CRISPR‐Cas12a‐based POCT technologies.

## 4 Experimental Section

The Materials and methods can be found in the supporting information.

## Funding

The National Key Research and Development Plan (No. 2023YFC2705400), the National Natural Science Foundation project (Nos. 82472965 and W2521097), and the Hubei Provincial Central Government‐Guided Local Science and Technology Fund Development Project (2024EIA002). The Hubei Provincial National Natural Science Foundation (2025AFD286). Free Innovation Pre‐research Fund of Union Hospital (2025XHYN047).

## Conflicts of Interest

The authors declare no conflicts of interest.

## Supporting information




**Supporting File**: advs74728‐sup‐0001‐SuppMat.docx.

## Data Availability

The data that support the findings of this study are available from the corresponding author upon reasonable request.
