# Supplementary material for: A Single‐Enzyme Activated CRISPR‐Cas12a Nano System via Subtly Balanced dsDNA for Kinetic‐Gated UDG Detection and Spatiotemporal Cellular Imaging
Source: Adv Sci (Weinh). 2026 Mar 7;13(28):e23400. doi: 10.1002/advs.202523400 (PMC13185816; doi:10.1002/advs.202523400)
Supplement: Supplementary file 1 — Supporting File: advs74728‐sup‐0001‐SuppMat.docx. [file ADVS-13-e23400-s001.docx]

Supporting Information

**A Single-Enzyme Activated CRISPR-Cas12a Nano System via Subtly Balanced dsDNA for Kinetic-Gated UDG Detection and Spatiotemporal Cellular Imaging**

*Kejun Dong, Hao Hu, Haiyun Wang, Zijia Zheng, Shuangshuang Cheng, Wan Shu, Ruisi Liu, Xiaoyan Xin, Shiyi Huang, Dingchao Qian, Xianjin Xiao* , Qiang Fu* and Hongbo Wang**

K-J Dong, H Hao, H-Y Wang, Z-J Zheng, S-S Cheng, W Shu, R-S Liu, X-Y Xin, X-J Xiao, H-B Wang

Department of Obstetrics and Gynecology, Union Hospital and Institute of Reproductive Health, Tongji Medical College, Huazhong University of Science and Technology, Wuhan China
E-mail: xiaoxianjin@hust.edu.cn, hb_wang1969@sina.com

S-Y Huang, D-C Qian, Qiang F

Department of Cardiology, South China Hospital, Medical School, Shenzhen University

Shenzhen, China

E-mail: fuqiang020@126.com

1. **Experimental Section**

*1.1 Materials*

The oligonucleotide strands were purchased from Sangon (Shanghai, China), the information were demonstrated in Table S1. The Uracil-DNA Glycosylase (UDG), Lba Cas12a, Bst 2.0 and Fpg were purchased from New England Biolads (USA). The Endo IV was purchased from Thermol Fisher (USA).The Hela cell line was purchased from Zhongqiaoxinzhou (Shanghai, China).

*1.2 UDG detection assays*

The Nanodrop 2000 (USA) was used to determine the concentration of the oligonucleotide strands. The UDG was added to the dsDNA substrate which was heating and annealing in advanced. The system was incubated under 37 ℃. 1x RNP was prepared for the assay with 500nM of the Cas12a, and 250nM of the crRNA strand. The100nM dsDNA substrate, 200 nM of the fluorescent probe, 5 ul of 10x NEB Buffer r2.1, 4 mM of Mg^2+^ were used for the detection. And it the deionized water was added to make the volume to 50ul. The system was sent to BioTek Synergy HTX (USA) for fluorescence detection. The linear section for fluorescence curve raising rate was used for calculation.

*1.3 Gel electrophoresis analysis*

The solutions was mixed with 6x loading buffer. The Tris-Glycine PAGE was used for gel electrophoresis analysis under 110v.

*1.4 Cell culture*

The Hela cells were incubated by 1640 medium (Servicebio, China) with 10% FBS under 37℃, 5% CO_2_. The cells lysate was obtained by the previous methods under the amount of 10^6^ per mL. The lysate was diluted in different concentration and was used for detection of the UDG assays.

*1.5 Intracellular Imaging*

The Cas12a-NLS-U6-sgRNA plasmid was constructed by Sangon Bio and the information was demonstrated in Table S4. The cells were seeded in the 24-well plate in advanced. The plasmid, the dsDNA substrate and the fluorescent probe were constructed to lipo nano-vehicle under the guide of Lipo8000 (Beyotime, China) and transfected to the cells. The fluorescent was observed by the microscope (Ningbo shunyu Optical Technology Co., Ltd) under the 470nm wavelength of the excitation light.

*1.6 Bio-information analysis*

Analysis of APE1 and UDG expression levels across different time points was performed using the GEPIA online tool to analyze tumor data from the TCGA. For survival analysis, the Kaplan-Meier Plotter was employed to examine patient survival outcomes associated with UNG (UDG) expression levels in the target tumor types.

*1.7 UGI screening assays*

The UGI was purchased from New England Bio (USA), and diluted into different concentrations. The UGI was incubated with UDG and the cell lysate at 37℃, and used the method of UDG assays to evaluate the inhibit effect.

*1.8 Cell cycle assays*

For the experimental group, cells were treated with serum-free medium. For cell cycle analysis, collect cells according to the protocol (Vazyme Biotech, China), then treat them with propidium iodide and cell permeabilization solution in the dark for 1 hour. Detect red fluorescence at an excitation wavelength of 488 nm using a flow cytometer.

*1.9 Simulation calculation*

Simulate the process of chain migration reactions using DNA strands. Analyze the free energy of the reaction's initial and final states using the NUPACK online analysis system.

*1.10 Statistic analysis*

The statistic analysis was used Graphpad Prism (8.0). P-value under 0.05 was considered with the significant difference. Unless otherwise specified, the number of replicate samples is 3.

*1.11 qRT-PCR analysis*

After cell processing, mRNA was extracted using the Trizol method. Following the reverse transcription kit instructions, mRNA was reverse transcribed into cDNA. Fluorescent PCR was performed in a Taq Mix system using SYBR Green dye. Ct values for each group were recorded and analyzed using the 2^-ΔΔCt^ method.

1. **Supplemental Table**

Table S1 The Oligonucleotide Chain Sequence (Fig.2)

| **Name** | **Sequence** |
| --- | --- |
| NTS-1 | GTGGAAGGATAACATATTTATGCTCTCTCTCTGGTTTGAGCGT |
| NTS-Simulator | GTGGAAGGATAACATAAAAATGCTCTCTCTCTGGTTTGAGCGT |
| NTS-Normal | GTGGAAGGATAACATATTTATGGGTTTGAGCGT |
| NTS-U | GTGGAAGGATAACATAUUUATGCTCTCTCTCTGGTTTGAGCGT |
| TS-1 | GCAGCTATGCTCACCACTATACCACCAACGCTCAAACCCATAAATATGTTATCCTTCCAC |
| crRNA | UAAUUUCUACUAAGUGUAGAUUAUACAUA UUUAUGGGUUUG |
| Probe | FAM-TTATTATT-BHQ |

Table S2 The Oligonucleotide Chain Sequence (Fig.3)

| **Name** | **Sequence** |
| --- | --- |
| NTS-10nt | GTGGAAGGATAACATATTTATGCTCTCTCTCTGGTTTGAGCGT |
| NTS-8nt | GTGGAAGGATAACATATTTATGGGCTCTCTCTTTTGAGCGT |
| NTS-6nt | GTGGAAGGATAACATATTTATGGGCTCTCTTTTGAGCGT |
| NTS-4nt | GTGGAAGGATAACATATTTATGGGCTCTTTTGAGCGT |
| NTS-10nt simulator | GTGGAAGGATAACATAAAAATGGGCTCTCTCTCTTTTGAGCGT |
| NTS-8nt simulator | GTGGAAGGATAACATAAAAATGGGCTCTCTCTTTTGAGCGT |
| NTS-6nt simulator | GTGGAAGGATAACATAAAAATGGGCTCTCTTTTGAGCGT |
| NTS-4nt simulator | GTGGAAGGATAACATAAAAATGGGCTCTTTTGAGCGT |
| NTS-position 2 | GTGGAAGGATAACATATTTATGGGTTCTCTCTCTCTTGAGCGT |
| NTS-position 6 | GTGGAAGGATAACATATTTATGCTCTCTCTCTGGTTTGAGCGT |
| NTS-position 8 | GTGGAAGGATAACATATTTACTCTCTCTCTTGGGTTTGAGCGT |
| NTS-position 10 | GTGGAAGGATAACATATTCTCTCTCTCTTATGGGTTTGAGCGT |
| crRNA mimics | TAATTTCTACTAAGTGTAGATTATACATA TTTATGGGTTTG |
| crRNA | UAAUUUCUACUAAGUGUAGAUUAUACAUA UUUAUGGGUUUG |

Table S3 The Oligonucleotide Chain Sequence (Fig.4)

| **Name** | **Sequence** |
| --- | --- |
| NTS-UUU | GTGGAAGGATAACATAUUUATGCTCTCTCTCTGGTTTGAGCGT |
| NTS-TUU | GTGGAAGGATAACATATUUATGCTCTCTCTCTGGTTTGAGCGT |
| NTS-TTU | GTGGAAGGATAACATATTUATGCTCTCTCTCTGGTTTGAGCGT |
| NTS-UTT | GTGGAAGGATAACATAUTTATGCTCTCTCTCTGGTTTGAGCGT |
| NTS-UTU | GTGGAAGGATAACATAUTUATGCTCTCTCTCTGGTTTGAGCGT |
| crRNA | UAAUUUCUACUAAGUGUAGAUUAUACAUA UUUAUGGGUUUG |

Table S4 The Key Sequence of the plasmid

| **Name** | **Sequence** |
| --- | --- |
| Cas12a | atgagcaagctggagaagtttacaaactgctactccctgtctaagaccctgaggttcaaggccatccctgtgggcaagacccaggagaacatcgacaataagcggctgctggtggaggacgagaagagagccgaggattataagggcgtgaagaagctgctggatcgctactatctgtcttttatcaacgacgtgctgcacagcatcaagctgaagaatctgaacaattacatcagcctgttccggaagaaaaccagaaccgagaaggagaataaggagctggagaacctggagatcaatctgcggaaggagatcgccaaggccttcaagggcaacgagggctacaagtccctgtttaagaaggatatcatcgagacaatcctgccagagttcctggacgataaggacgagatcgccctggtgaacagcttcaatggctttaccacagccttcaccggcttctttgataacagagagaatatgttttccgaggaggccaagagcacatccatcgccttcaggtgtatcaacgagaatctgacccgctacatctctaatatggacatcttcgagaaggtggacgccatctttgataagcacgaggtgcaggagatcaaggagaagatcctgaacagcgactatgatgtggaggatttctttgagggcgagttctttaactttgtgctgacacaggagggcatcgacgtgtataacgccatcatcggcggcttcgtgaccgagagcggcgagaagatcaagggcctgaacgagtacatcaacctgtataatcagaaaaccaagcagaagctgcctaagtttaagccactgtataagcaggtgctgagcgatcgggagtctctgagcttctacggcgagggctatacatccgatgaggaggtgctggaggtgtttagaaacaccctgaacaagaacagcgagatcttcagctccatcaagaagctggagaagctgttcaagaattttgacgagtactctagcgccggcatctttgtgaagaacggccccgccatcagcacaatctccaaggatatcttcggcgagtggaacgtgatccgggacaagtggaatgccgagtatgacgatatccacctgaagaagaaggccgtggtgaccgagaagtacgaggacgatcggagaaagtccttcaagaagatcggctccttttctctggagcagctgcaggagtacgccgacgccgatctgtctgtggtggagaagctgaaggagatcatcatccagaaggtggatgagatctacaaggtgtatggctcctctgagaagctgttcgacgccgattttgtgctggagaagagcctgaagaagaacgacgccgtggtggccatcatgaaggacctgctggattctgtgaagagcttcgagaattacatcaaggccttctttggcgagggcaaggagacaaacagggacgagtccttctatggcgattttgtgctggcctacgacatcctgctgaaggtggaccacatctacgatgccatccgcaattatgtgacccagaagccctactctaaggataagttcaagctgtattttcagaaccctcagttcatgggcggctgggacaaggataaggagacagactatcgggccaccatcctgagatacggctccaagtactatctggccatcatggataagaagtacgccaagtgcctgcagaagatcgacaaggacgatgtgaacggcaattacgagaagatcaactataagctgctgcccggccctaataagatgctgccaaaggtgttcttttctaagaagtggatggcctactataaccccagcgaggacatccagaagatctacaagaatggcacattcaagaagggcgatatgtttaacctgaatgactgtcacaagctgatcgacttctttaaggatagcatctcccggtatccaaagtggtccaatgcctacgatttcaacttttctgagacagagaagtataaggacatcgccggcttttacagagaggtggaggagcagggctataaggtgagcttcgagtctgccagcaagaaggaggtggataagctggtggaggagggcaagctgtatatgttccagatctataacaaggacttttccgataagtctcacggcacacccaatctgcacaccatgtacttcaagctgctgtttgacgagaacaatcacggacagatcaggctgagcggaggagcagagctgttcatgaggcgcgcctccctgaagaaggaggagctggtggtgcacccagccaactcccctatcgccaacaagaatccagataatcccaagaaaaccacaaccctgtcctacgacgtgtataaggataagaggttttctgaggaccagtacgagctgcacatcccaatcgccatcaataagtgccccaagaacatcttcaagatcaatacagaggtgcgcgtgctgctgaagcacgacgataacccctatgtgatcggcatcgataggggcgagcgcaatctgctgtatatcgtggtggtggacggcaagggcaacatcgtggagcagtattccctgaacgagatcatcaacaacttcaacggcatcaggatcaagacagattaccactctctgctggacaagaaggagaaggagaggttcgaggcccgccagaactggacctccatcgagaatatcaaggagctgaaggccggctatatctctcaggtggtgcacaagatctgcgagctggtggagaagtacgatgccgtgatcgccctggaggacctgaactctggctttaagaatagccgcgtgaaggtggagaagcaggtgtatcagaagttcgagaagatgctgatcgataagctgaactacatggtggacaagaagtctaatccttgtgcaacaggcggcgccctgaagggctatcagatcaccaataagttcgagagctttaagtccatgtctacccagaacggcttcatcttttacatccctgcctggctgacatccaagatcgatccatctaccggctttgtgaacctgctgaaaaccaagtataccagcatcgccgattccaagaagttcatcagctcctttgacaggatcatgtacgtgcccgaggaggatctgttcgagtttgccctggactataagaacttctctcgcacagacgccgattacatcaagaagtggaagctgtactcctacggcaaccggatcagaatcttccggaatcctaagaagaacaacgtgttcgactgggaggaggtgtgcctgaccagcgcctataaggagctgttcaacaagtacggcatcaattatcagcagggcgatatcagagccctgctgtgcgagcagtccgacaaggccttctactctagctttatggccctgatgagcctgatgctgcagatgcggaacagcatcacaggccgcaccgacgtggattttctgatcagccctgtgaagaactccgacggcatcttctacgatagccggaactatgaggcccaggagaatgccatcctgccaaagaacgccgacgccaatggcgcctataacatcgccagaaaggtgctgtgggccatcggccagttcaagaaggccgaggacgagaagctggataaggtgaagatcgccatctctaacaaggagtggctggagtacgcccagaccagcgtgaagcac |

Table S5 The primer sequence

| **Name** | **Sequence** |
| --- | --- |
| ACTB FP | GTACCACTGGCATCGTGATGGACT |
| ACTB RP | CCGCTCATTGCCAATGGTGAT |
| UDG FP | TCTCCCCGCTCCAGTTTAGA |
| UDG RP | GCAGAAGACGCCCATTTGTG |

Table S6 Representative recent methods for UDG detection

| **Method** | **Design Principle** | **Assay Time** | **LOD(U/mL)** | **Features** | **Reference** |
| --- | --- | --- | --- | --- | --- |
| CRISPR-Cas12a +Endo IV cascade | UDG+ APE1/Endo-processing generates Cas activator | 30-100min | 2.5x10^-6^ | Multi-enzyme cascade | [1] |
| Isothermal DNA repair-retarded CRISPR amplification | DNA repair-mediated isothermal amplification | 50min | 9.17x10^-4^ | One-pot amplification | [2] |
| λ-Exonuclease fluorescent assay | UDG-induced structural change triggers λ-exo digestion | 1-2h | 10^-4^ | Enzyme-assisted readout | [3] |
| Distance-based paper device (dPAD) with DNA hydrogel | DNA network cleavage | 80min | 6.4x10^-4^ | Instrument-free readout | [4] |
| Electrochemical nanozyme cascade amplification | UDG + nanozyme electrocatalytic amplification | 1h | 4.8x10^-4^ | Electrochemical detection | [5] |
| This work | Substrate kinetic gatekeeper directly converts uracil excision into Cas12a activation | 30-60min | 5x10^-7^ | Single-enzyme trigger; live-cell imaging | / |

Reference:

[1] K. Dong, W. Shu, J. Zhang, S. Cheng, J. Zhang, R. Zhao, T. Hua, W. Zhang, H. Wang, Biosensors and Bioelectronics 2023, 226, 115118, https://doi.org/https://doi.org/10.1016/j.bios.2023.115118.

[2] H. Han, S. H. Jang, J. K. Ahn, C. Y. Lee, Analytica Chimica Acta 2024, 1314, 342799, https://doi.org/https://doi.org/10.1016/j.aca.2024.342799.

[3] J. Sun, C. Li, Y. Hu, Y. Ding, T. Wu, Talanta 2022, 243, 123350, https://doi.org/https://doi.org/10.1016/j.talanta.2022.123350.

[4] W. Xue, Y. Wu, X. Li, Q. Zhang, Y. Wu, Y. Chang, M. Liu, Biosensors and Bioelectronics 2024, 264, 116687, https://doi.org/https://doi.org/10.1016/j.bios.2024.116687.

[5] T. Liu, Z. Li, M. Chen, H. Zhao, Z. Zheng, L. Cui, X. Zhang, Biosensors and Bioelectronics 2021, 194, 113607, https://doi.org/https://doi.org/10.1016/j.bios.2021.113607.

1. **Supplementary figures**


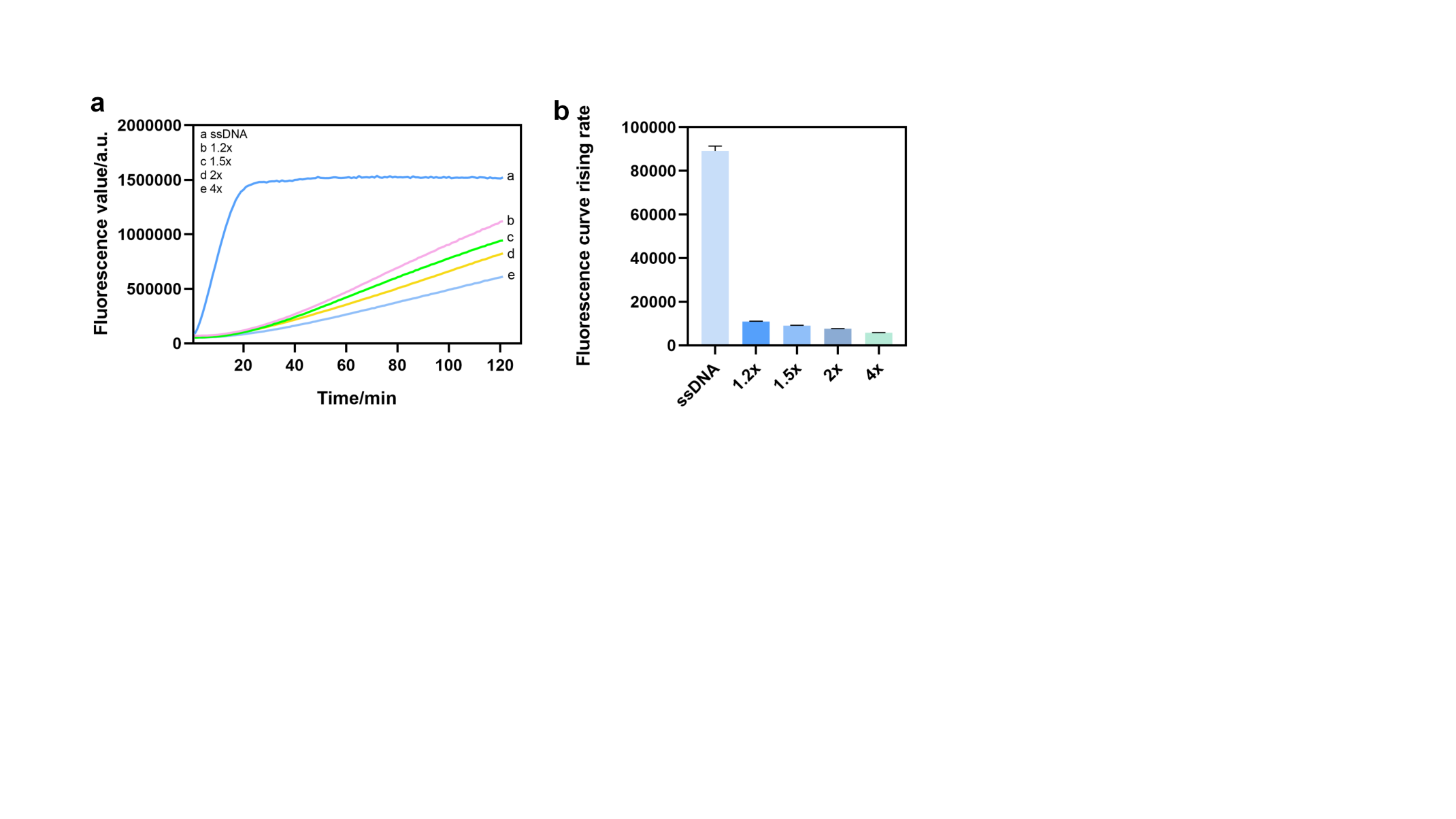


**Figure S1. The fluorescence curve of the dsDNA using NEB r2.1 buffer in the progress of heating and annealing.** (a,b) The comparison of different concentrations of NTS strand with the positive control.


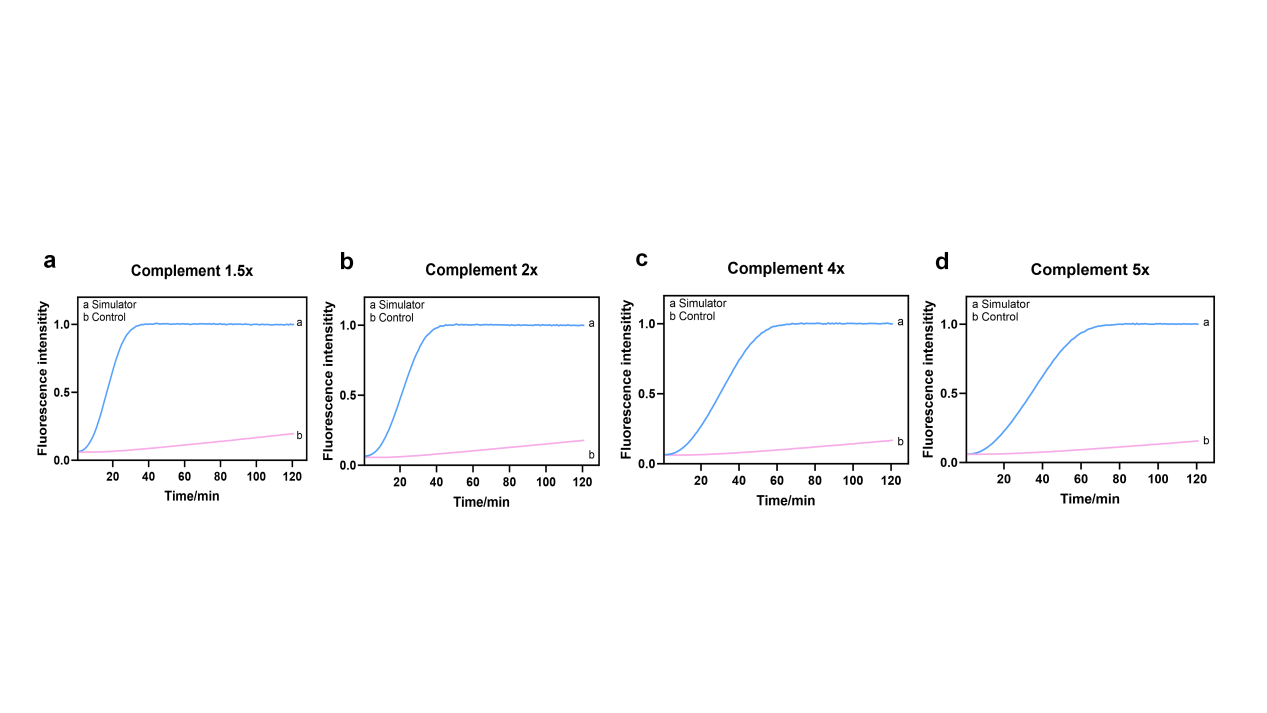


**Figure S2. Comparison of the different concentrations of NTS strand in simulation system.** (a-d) 1.5x, 2x, 4x, 5x.


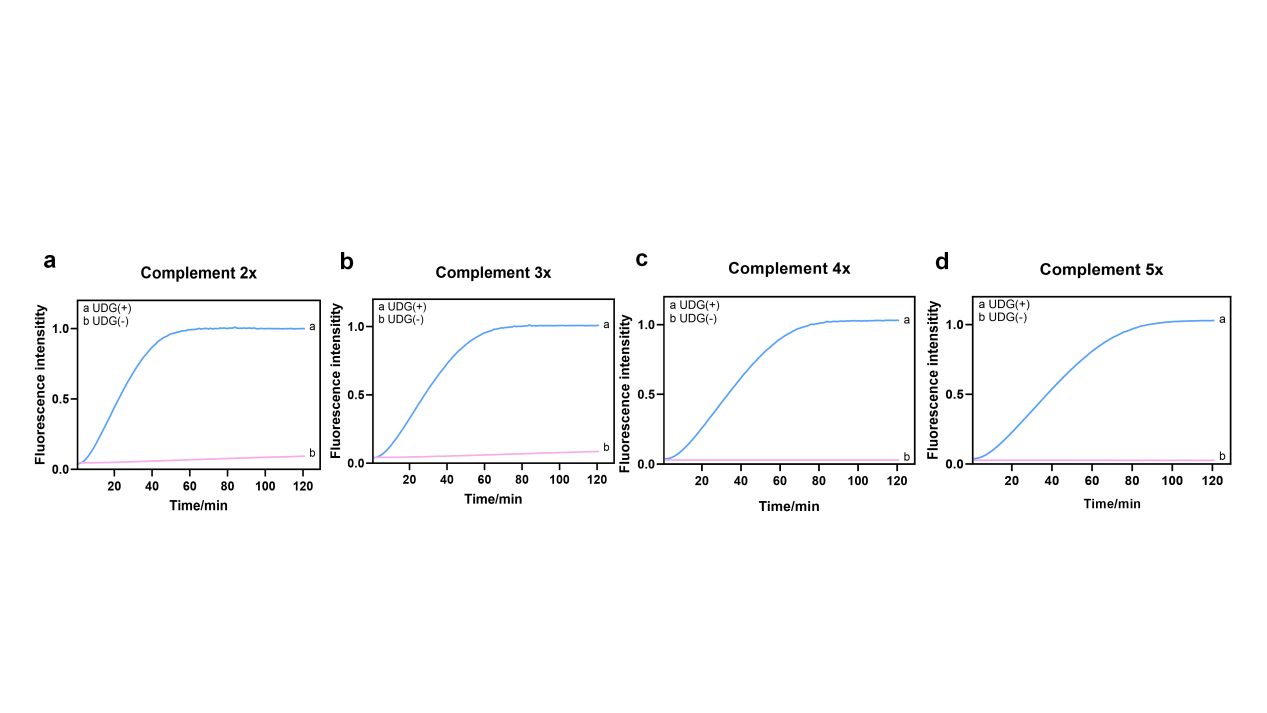


**Figure S3.** **Comparison of the different concentrations of NTS strand under UDG treatment.** (a-d) 1.5x, 2x, 4x, 5x.


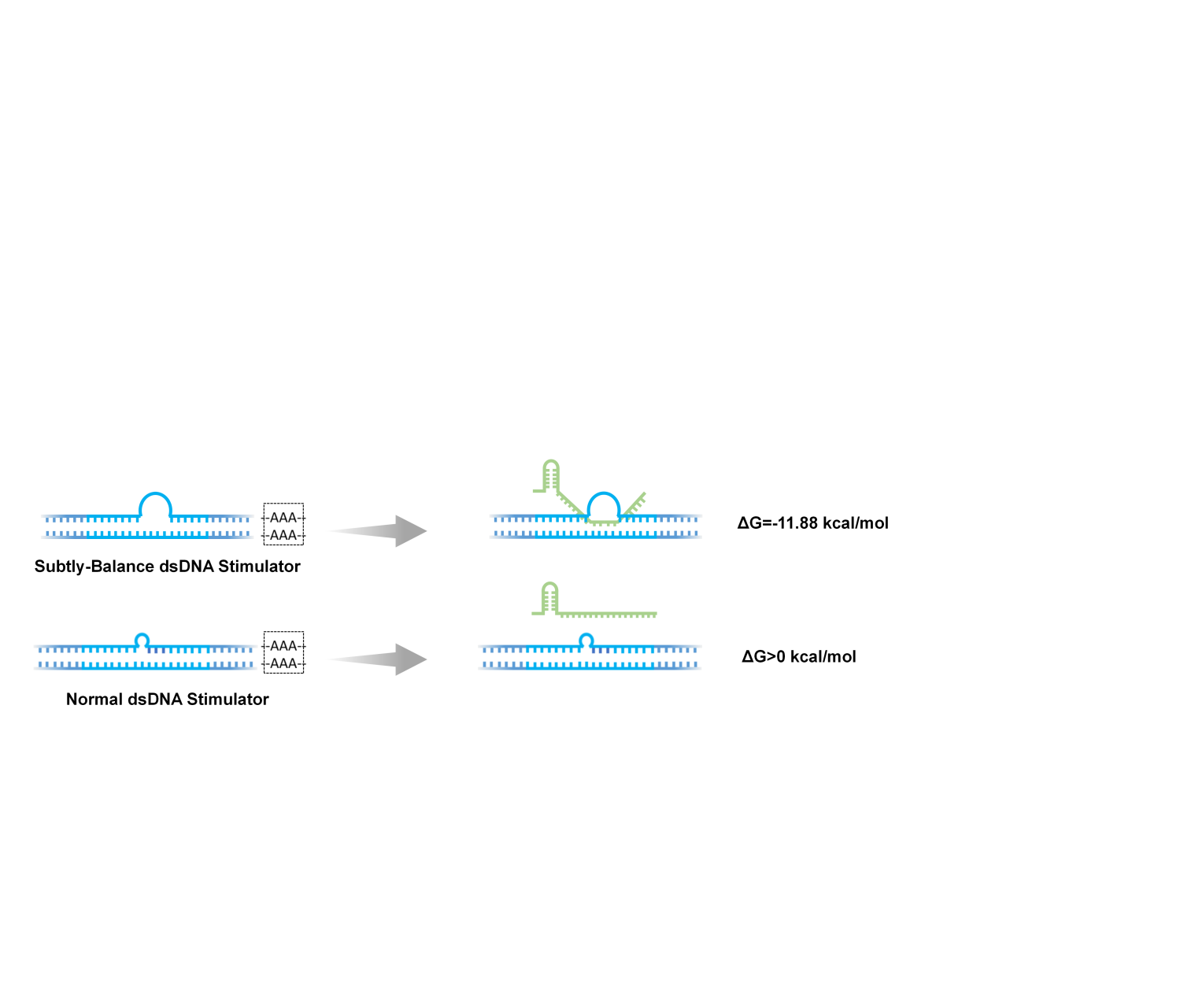


**Figure S4. Simulation calculation of the reaction by NUPACK.** Utilizing mismatch simulation for UDG processing. Compare the ΔG of hybridization reactions between normal dsDNA and subtly-balanced dsDNA with crRNA mimics.


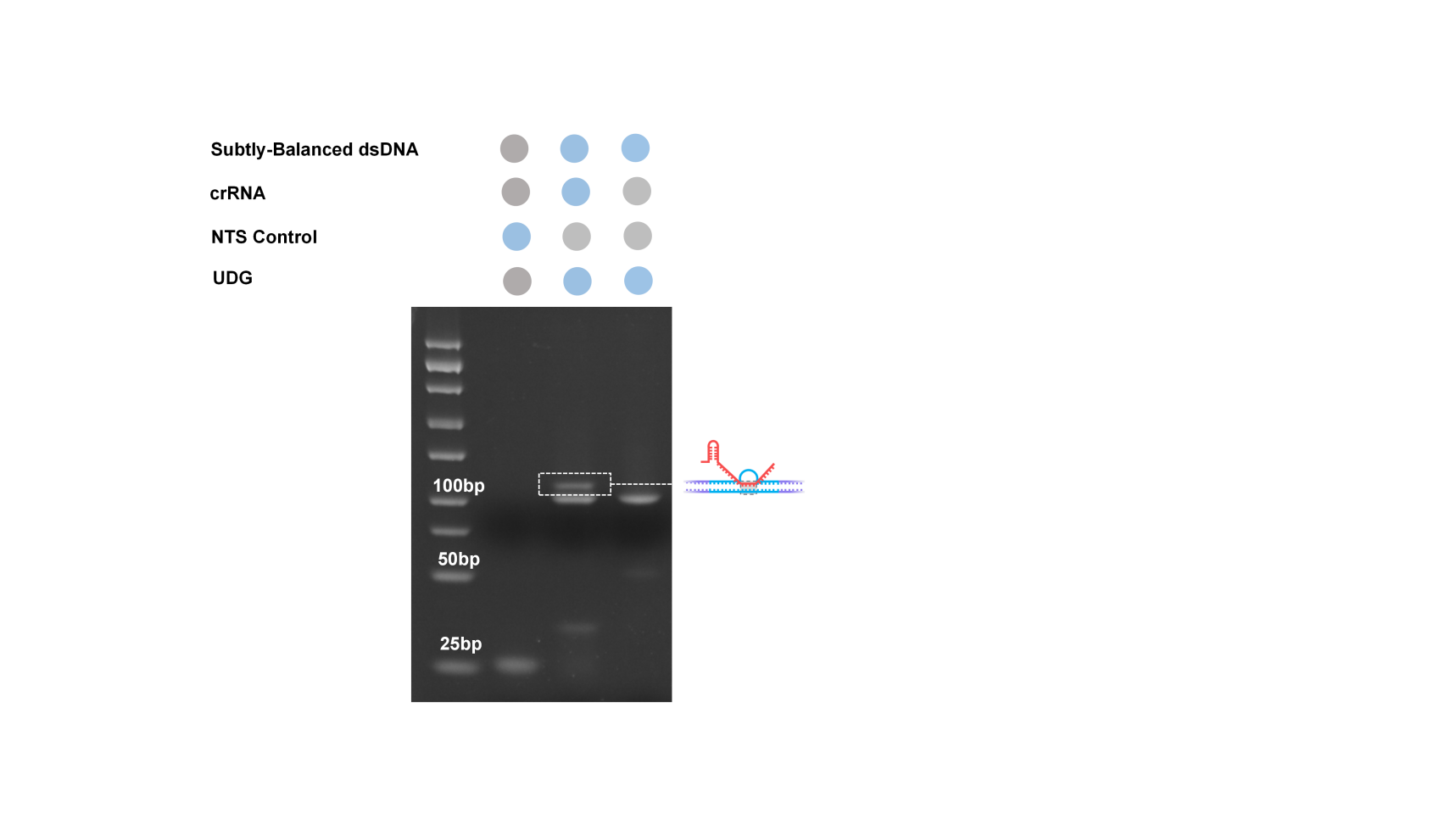


**Figure S5 Native PAGE electrophoresis analysis of crRNA reaction with subtly-balanced dsDNA substrate.**


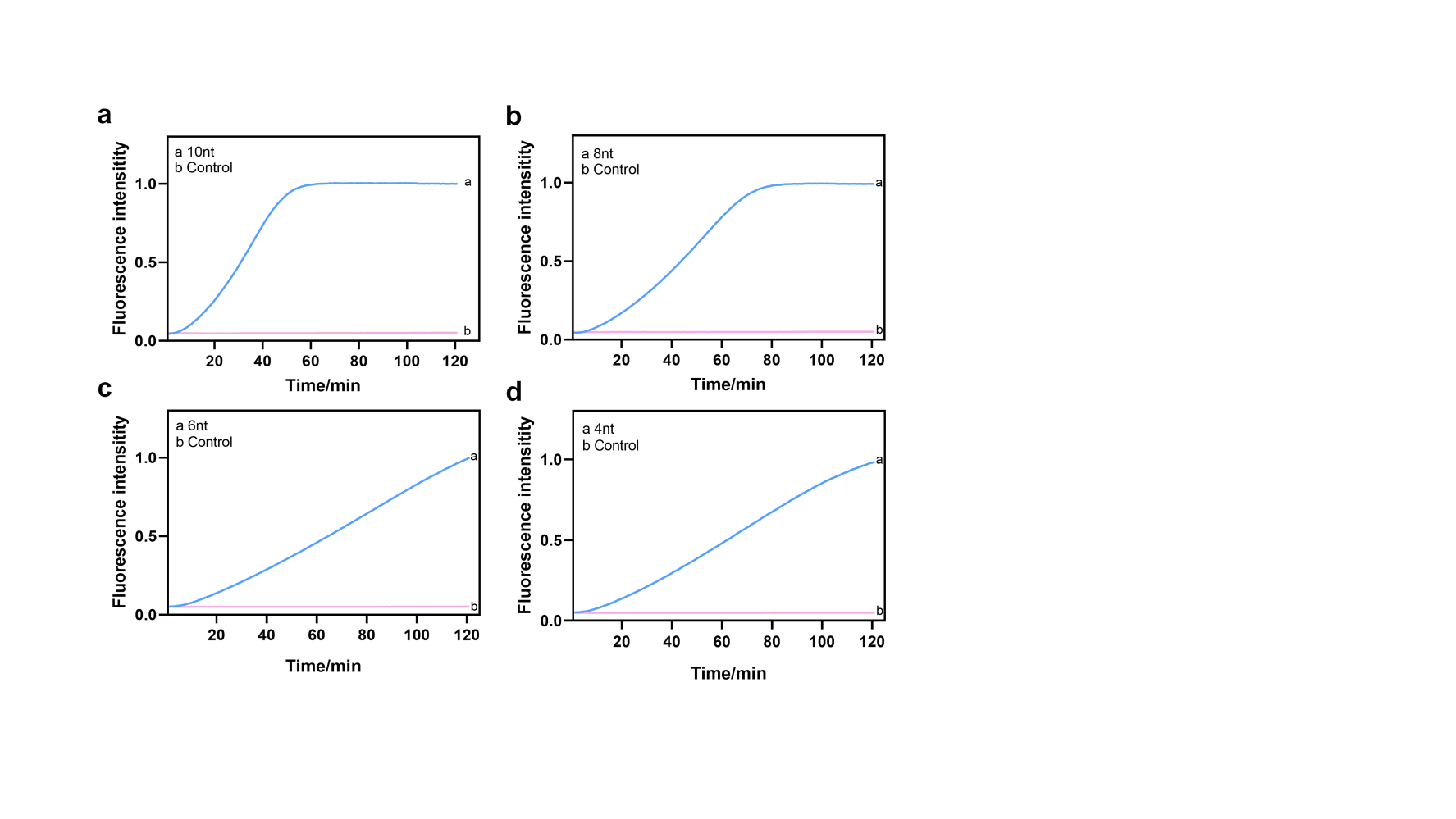


**Figure S6. Optimization of the bubble size.** (a-d) 10nt, 8nt, 6nt, 4nt. For the experimental group use the simulator NTS stand.


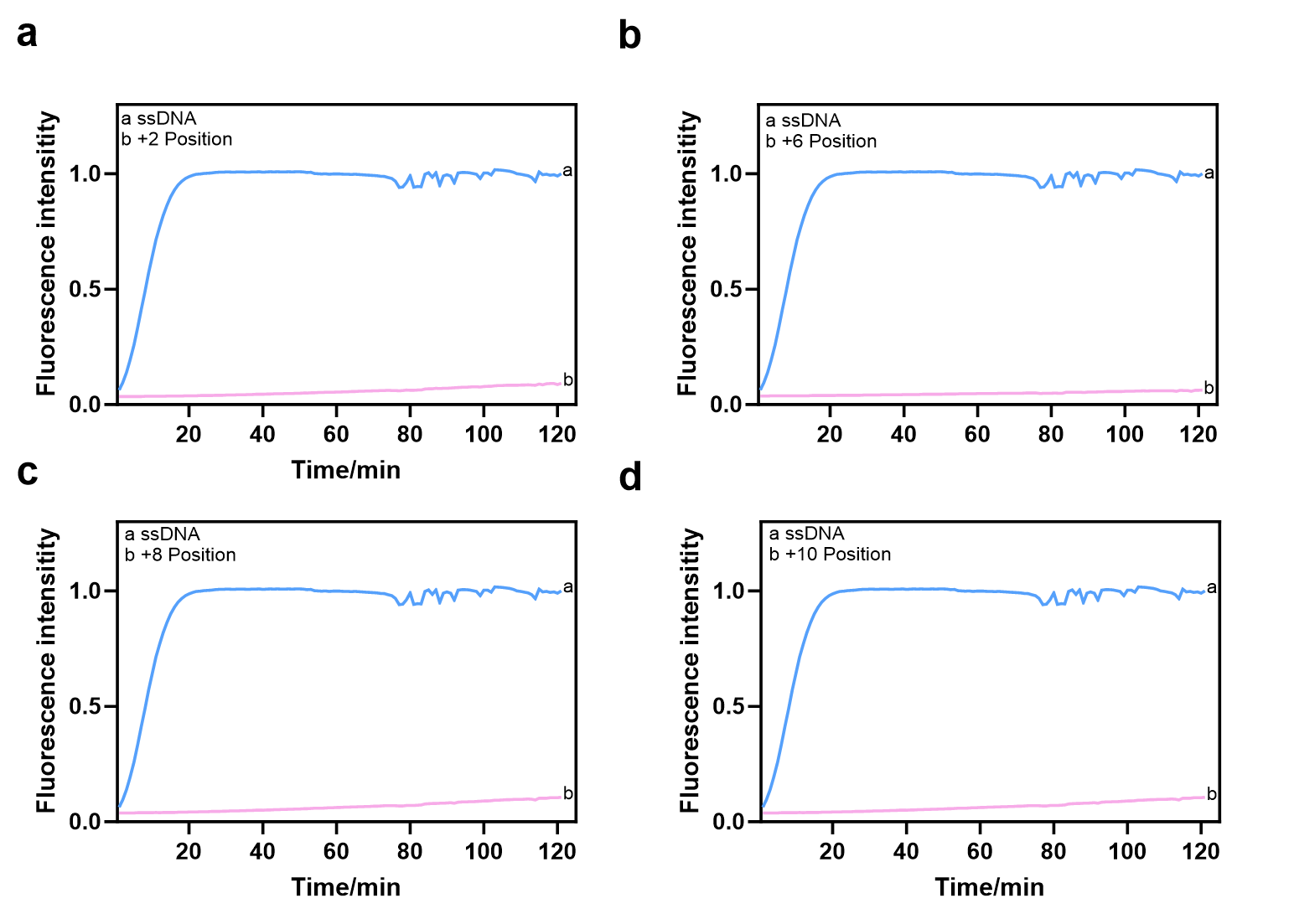


**Figure S7. The leakage evaluation of the different position of the bubble.** (a-d)Position No. +2, +6, +8, +10, compared with the positive control.


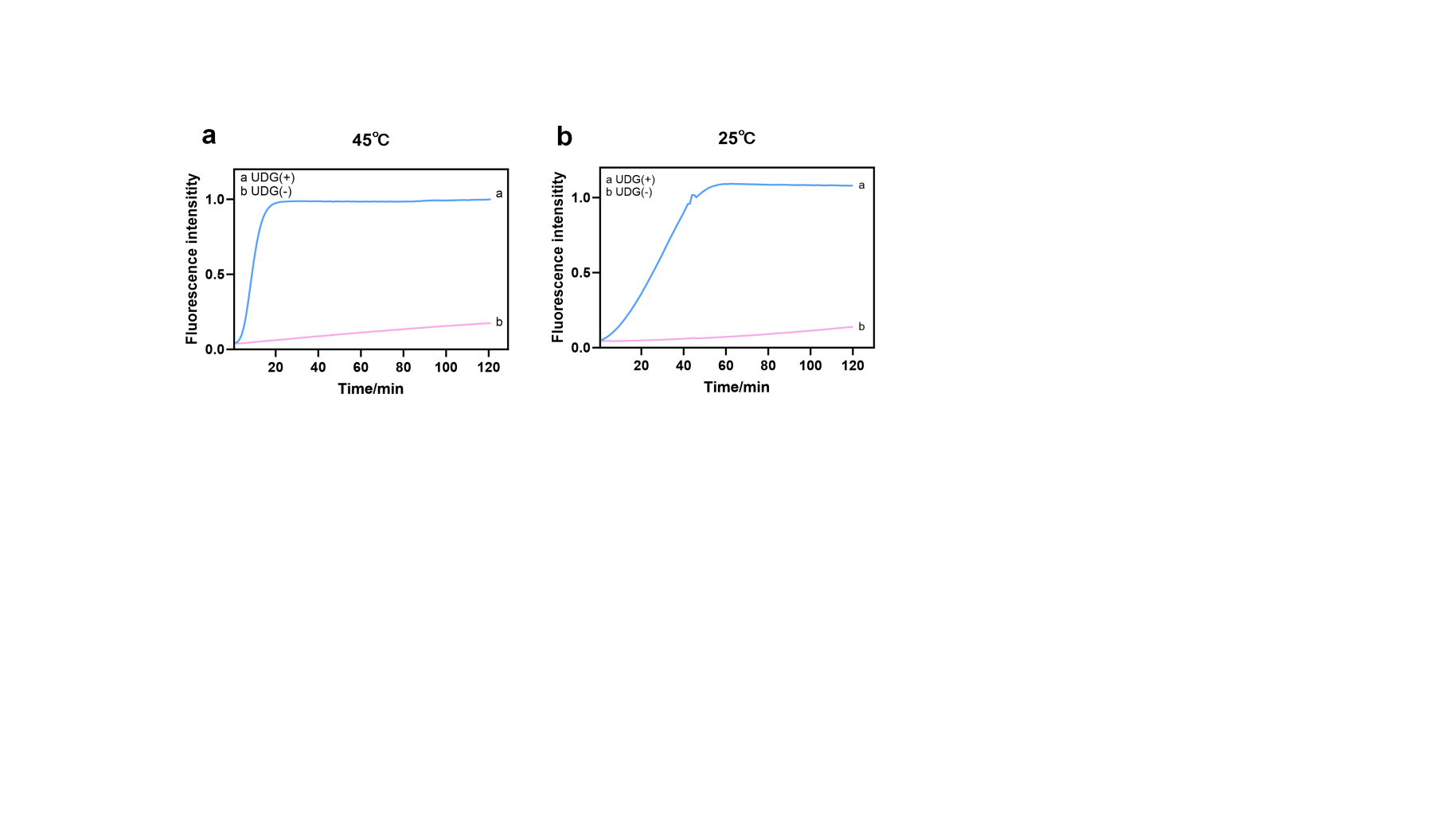


**Figure S8. Optimization of the temperature.** (a) 45°C, (b) 25°C .


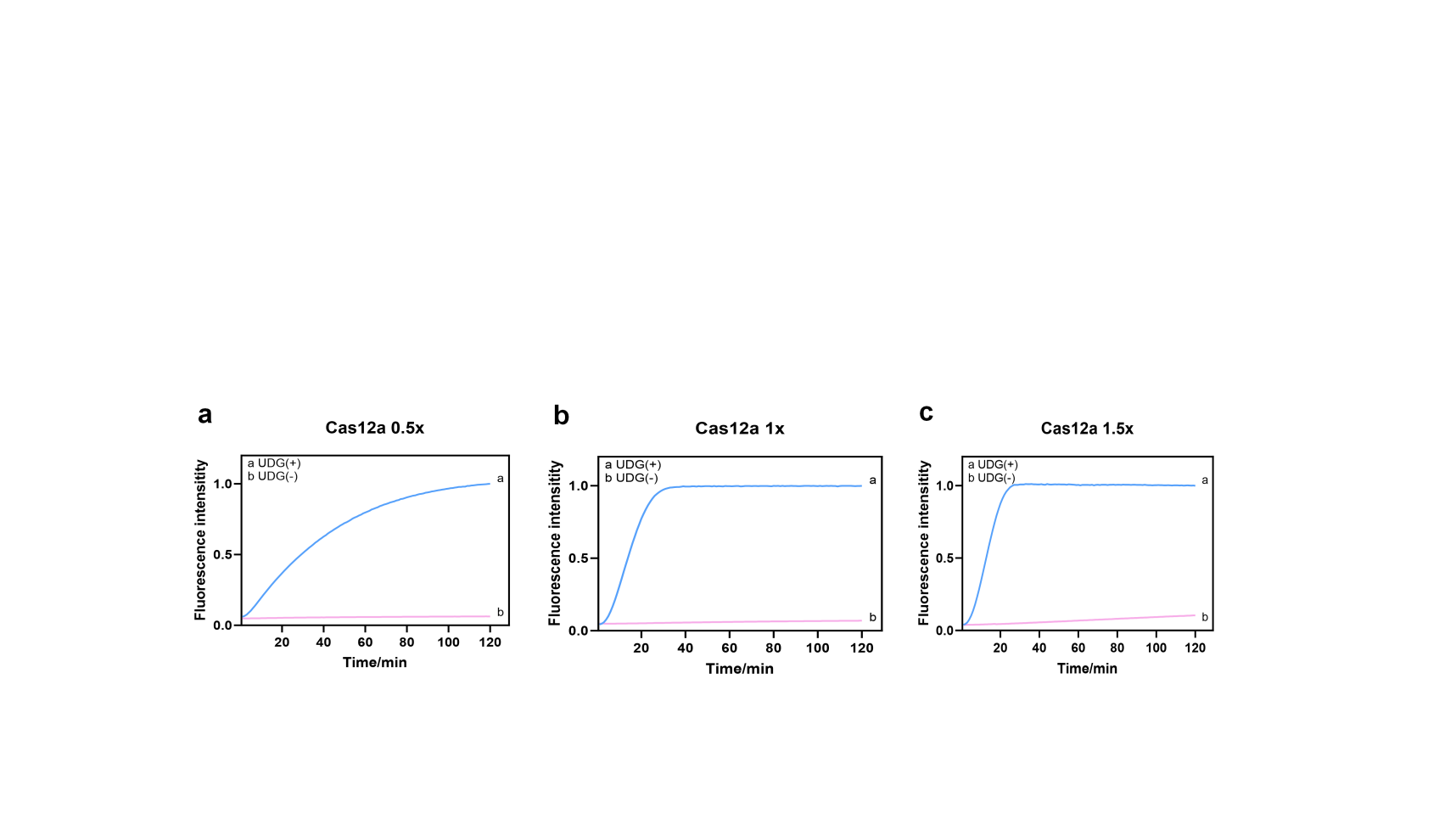


**Figure S9. Optimization of the concentrations of Cas12a RNP.** (a-c) 0.5x, 1x, 1.5x.


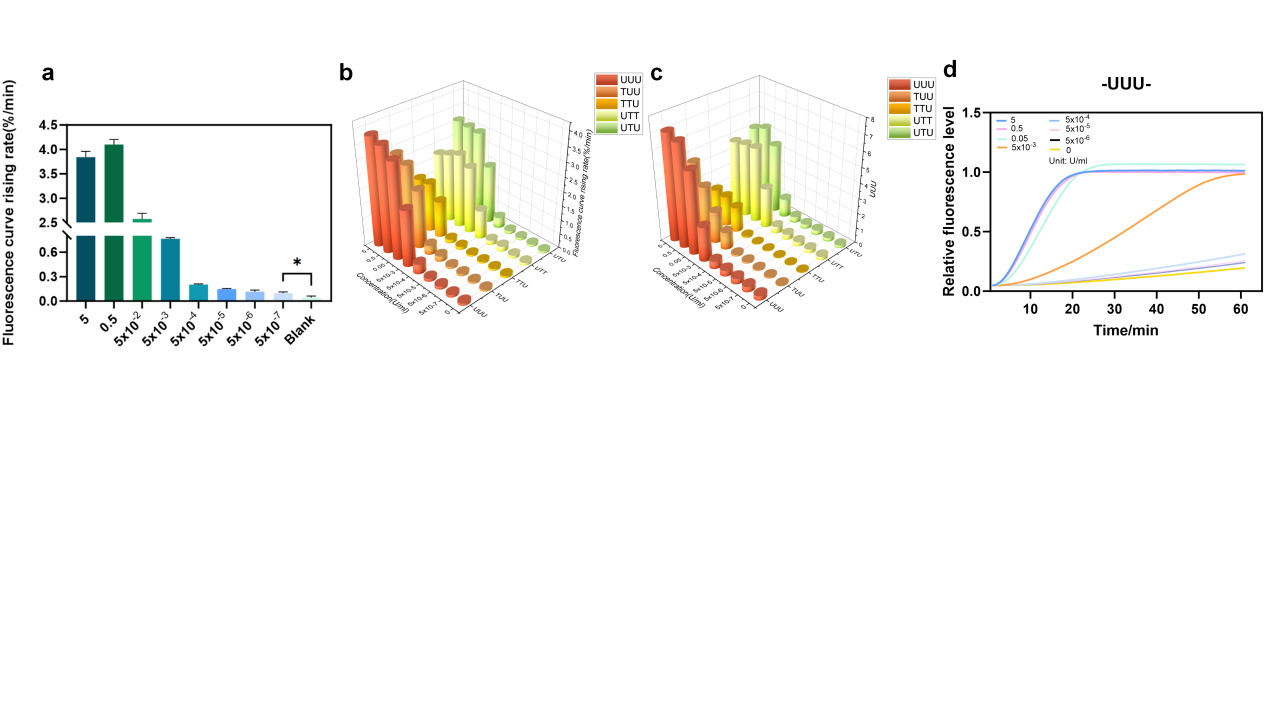


**Figure S10. Orthogonal Matrix Optimization of the system.** (a) The fluorescence curve rising rate for the group of UTU at RNP 1X. *, P=0.0178. n=3. (b) RNP concentration at 0.5x. (c) RNP concentration at 1.5x. (d) The system can detect 5x10-6 U/mL within 60 minutes at 1.5x RNP with UUU NTS strand.


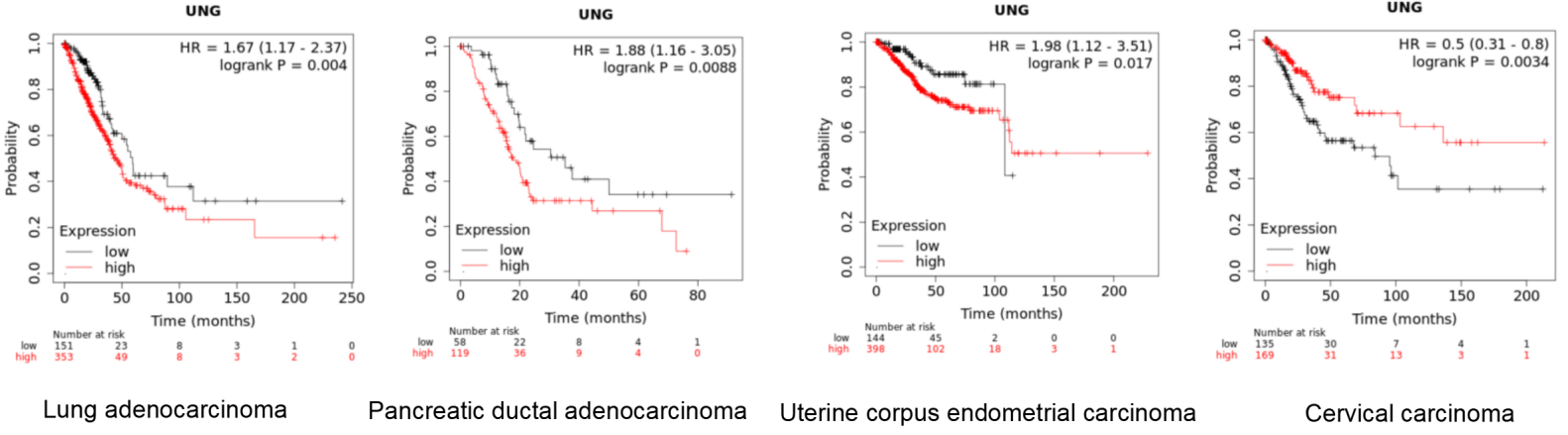


**Figure S11. The survival analysis of UDG level in different disease by the data of TCGA.**

**
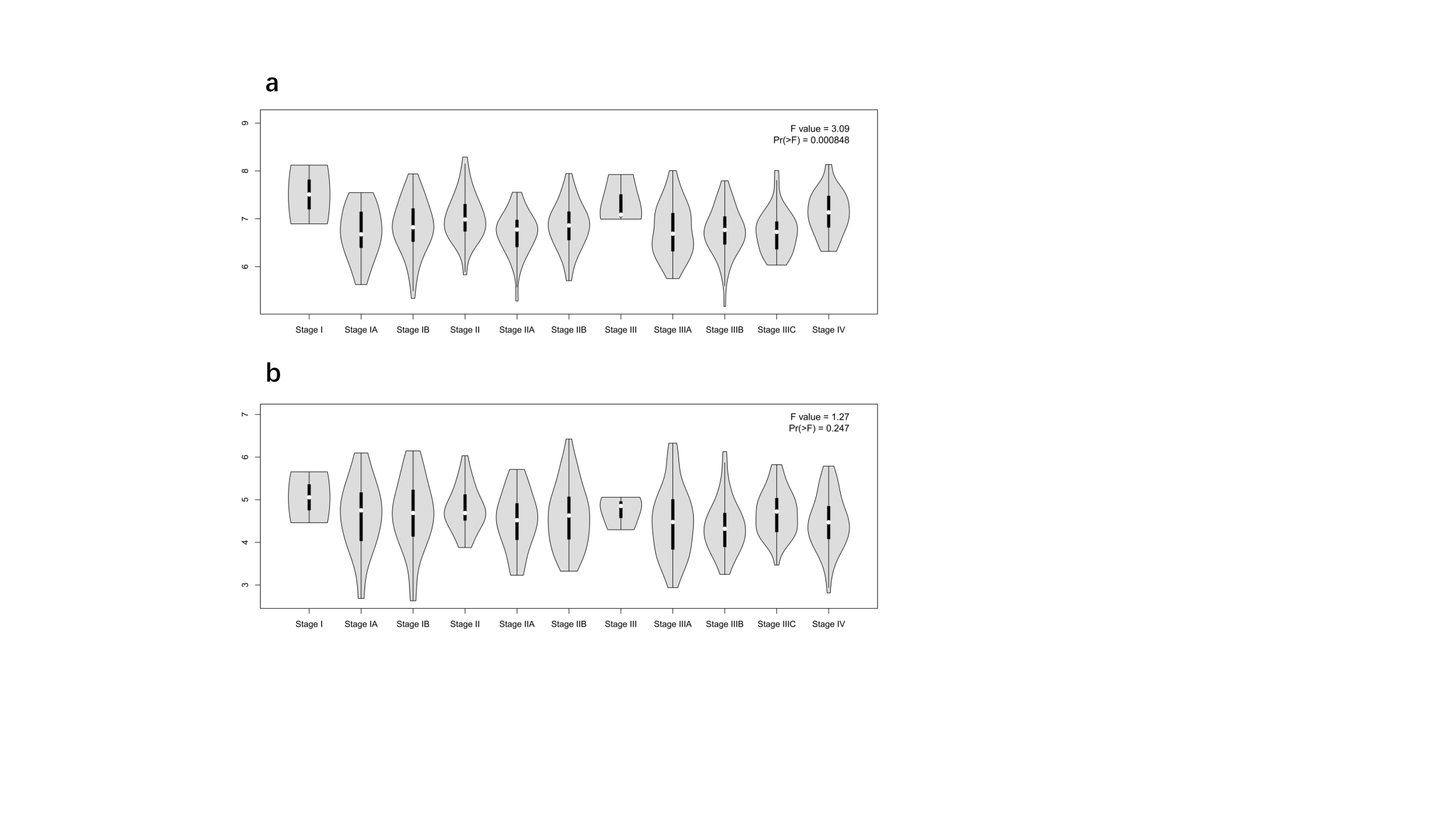
**

**Figure S12. Expression of UDG and APE1 in Stomach Adenocarcinoma Across Different Cell Cycle Phases.** (a) APE1 expression levels. Pr<0.05. (b) UDG expression levels, Pr>0.05.

**
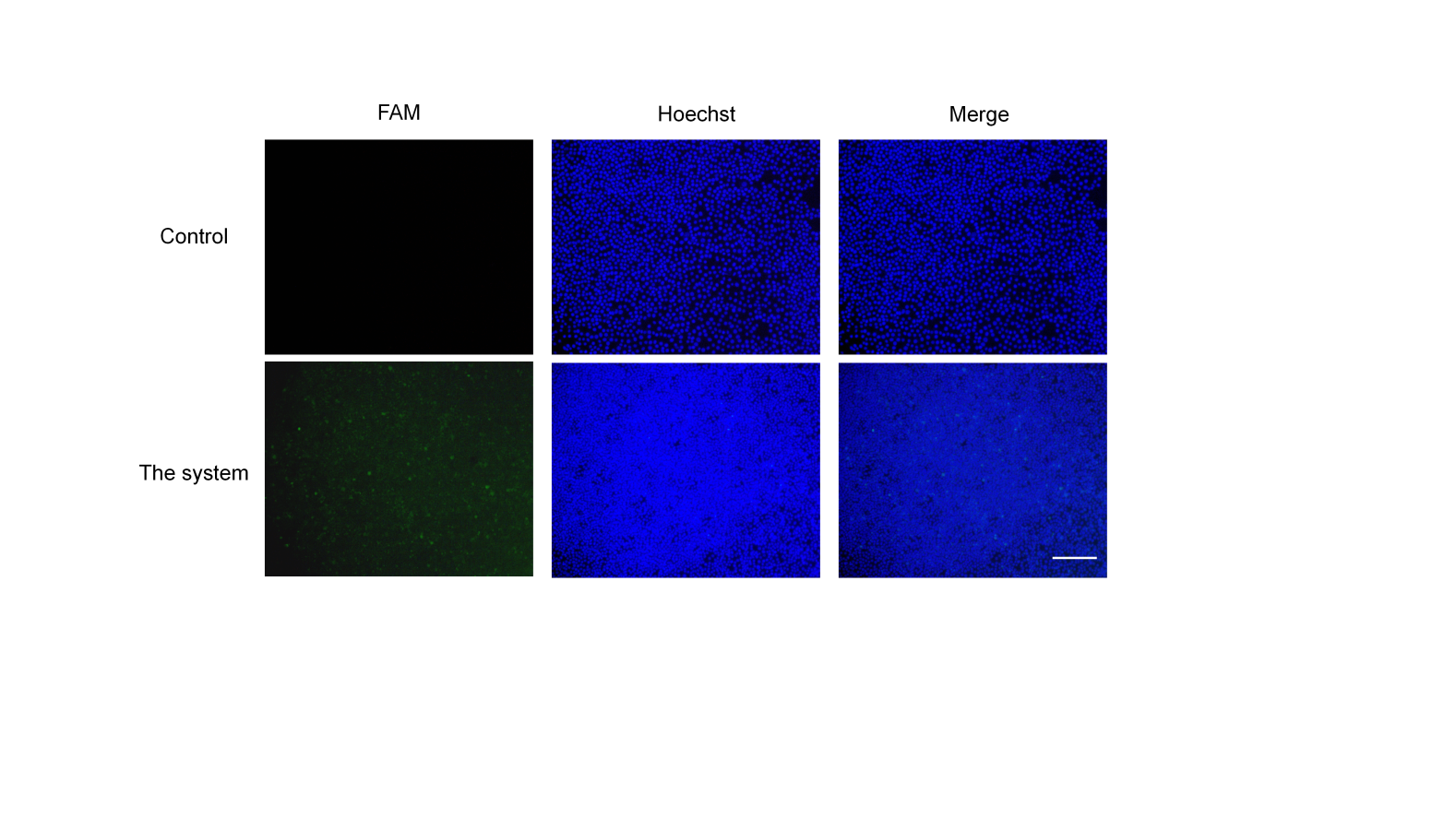
**

**Figure S13.** **The fluorescence photos for the evaluation of the delivery efficiency.** Use the FAM-probe instead of the FAM-BHQ probe. Scare bar, 50um.


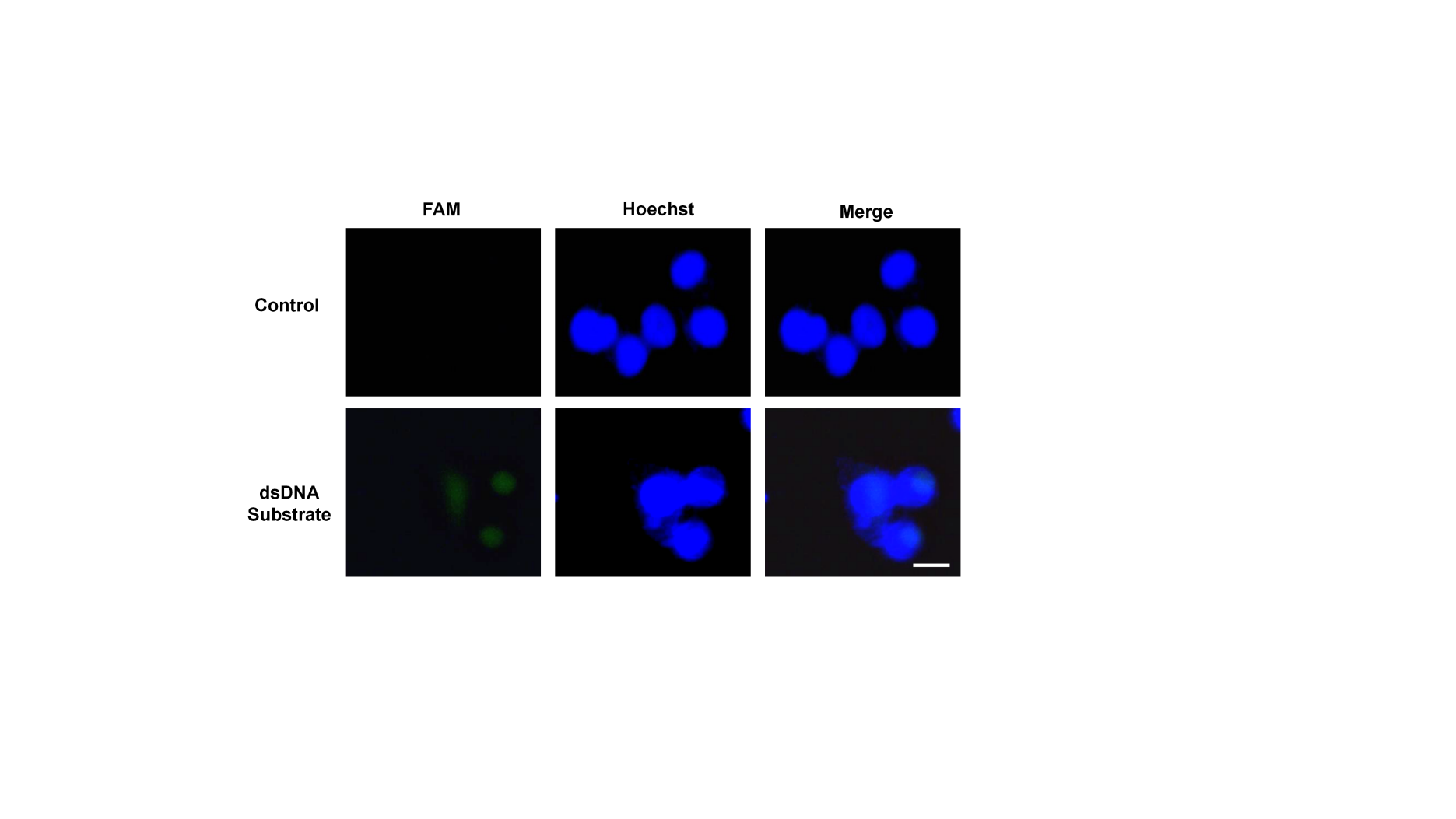


**Figure S14 Fluorescence analysis of FAM-labeled subtly-balance dsDNA substrates colocalizing with the cell nucleus. Scare bar, 10um.**
